# Integrated bulk RNA sequencing and mass cytometry analysis reveal the circulating immune landscape in ischemic and hemorrhagic Moyamoya disease

**DOI:** 10.1186/s12865-025-00699-3

**Published:** 2025-03-10

**Authors:** Chenglong Liu, Peicong Ge, Siqi Mou, Yuheng Pang, Liujia Chan, Junsheng Li, Qiheng He, Wei Liu, Bojian Zhang, Zhikang Zhao, Zhiyao Zheng, Shuang Wang, Wei Sun, Qian Zhang, Rong Wang, Yan Zhang, Wenjing Wang, Dong Zhang, Jizong Zhao

**Affiliations:** 1 Department of Neurosurgery, Beijing Tiantan Hospital, Capital Medical University, Beijing, 100070 China; 2 China National Clinical Research Center for Neurological Diseases, Beijing, 100070 China; 3 Medical School, University of Chinese Academy of Sciences, Beijing, 101408 China; 4 Beijing YouAn Hospital, Beijing Institute of Hepatology, Capital Medical University, Beijing, 100069 China; 5 Department of Medicinal Chemistry, College of Pharmaceutical Sciences of Capital Medical University, Beijing, 100069 P. R. China; 6 Department of Neurosurgery, Beijing Hospital, National Center of Gerontology, Beijing, 100730 China; 7 Institute of Geriatric Medicine, Chinese Academy of Medical Sciences, Beijing, 100730 China

**Keywords:** Moyamoya disease, Ischemia, Hemorrhage, Mass cytometry, Peripheral immune profiles

## Abstract

**Background:**

Moyamoya disease (MMD) is increasingly recognized as being influenced by chronic inflammation, with circulating immune cells playing a role in its progression. However, research on the immune characteristics of different MMD subtypes is limited. This study aims to compare the peripheral immune profiles of ischemic and hemorrhagic MMD patients.

**Methods:**

Peripheral immune profiles were analyzed using transcriptome sequencing and mass cytometry. Data preprocessing was followed by functional and gene set enrichment analyses, as well as the construction of immune-related gene sets and protein-protein interaction networks. High-dimensional data analysis was performed using the PhenoGraph and t-SNE algorithms.

**Results:**

The study involved 9 ischemic and 6 hemorrhagic MMD patients for transcriptome analysis, and 20 ischemic and 16 hemorrhagic patients for mass cytometry. Hemorrhagic MMD patients exhibited upregulated genes associated with inflammation, hypoxia, and bacterial responses and downregulated genes related to immune response regulation. The results of mass cytometry analysis showed that, compared to ischemic MMD, patients with hemorrhagic MMD had reduced CD3 expression levels in T cells and their specific subsets, as well as impaired chemotactic capacity of DPT cells. The function of the B03 subset in B cells was diminished, while the proportion of NK cells increased and that of monocytes decreased. Additionally, the proportions of the D03 and D07 subsets in dendritic cells (DCs) were elevated.

**Conclusions:**

This study reveals distinct immune profiles in ischemic and hemorrhagic MMD, emphasizing the need for subtype-specific therapeutic strategies.

**Supplementary Information:**

The online version contains supplementary material available at 10.1186/s12865-025-00699-3.

## Background

Moyamoya disease (MMD), a rare cerebrovascular disorder, is characterized by progressive stenosis or occlusion at the terminal portion of the internal carotid artery and the origins of the anterior and middle cerebral arteries, accompanied by the formation of abnormal vascular networks at the base of the brain [1]. In Asian populations, MMD typically presents in two phenotypes: ischemic MMD, commonly seen in pediatric patients, and hemorrhagic MMD, which predominantly affects adults. Patients with hemorrhagic MMD exhibit significantly higher disability and mortality rates compared to those with ischemic MMD, leading to poorer prognoses [2, 3]. Studies suggest that hemorrhagic MMD patients who undergo encephalo-duro-arterio-synangiosis (EDAS) show improved long-term outcomes and a reduced probability of rebleeding [4, 5].

Ischemic and hemorrhagic MMD exhibit significant differences in both pathological and clinical characteristics. The pathological features of ischemic MMD include ischemic damage resulting from arterial stenosis and occlusion, with inflammation playing a crucial role [6]. In contrast, hemorrhagic MMD is primarily characterized by the tendency of abnormal collateral vessels to rupture and bleed, which is associated with abnormal arterial branching and dilation [7, 8]. Studies suggest that dilation of the posterior communicating and anterior choroidal arteries in hemorrhagic MMD is associated with the initial hemorrhage but not with recurrent bleeding [9]. Additionally, high-resolution magnetic resonance imaging reveals more lipid deposits in the intracranial arterial walls of ischemic MMD patients, whereas hemorrhagic MMD patients exhibit fewer lipid deposits, suggesting distinct etiologies for each subtype [10, 11].

MMD is closely related to immunity and inflammation, with autopsy results showing T cell and macrophage infiltration in the affected vessels of MMD patients [12]. Recent studies indicate that peripheral immunity is closely associated with the pathogenesis of MMD, with an imbalance in T cell populations observed in MMD patients [13]. However, most research on MMD subtypes has focused on genetics and imaging [14], with few studies addressing the immune and peripheral circulation aspects. This study employs transcriptome sequencing and mass cytometry (a single-cell flow cytometry technique) to investigate the peripheral immune profiles of MMD subtypes, providing insights into the circulating immune landscapes of ischemic and hemorrhagic MMD, and exploring potential molecular therapeutic targets for hemorrhagic MMD.

## Methods

### Patients selection and data acquisition

Patients included in this study were diagnosed with MMD using digital subtraction angiography (DSA) based on the 2012 Japanese guidelines [15]. Patients with a history of cerebral hemorrhage were classified into the hemorrhagic MMD group, while those presenting with transient ischemic attacks (TIA) or cerebral infarction were assigned to the ischemic MMD group [16].

**Fig. 1:**
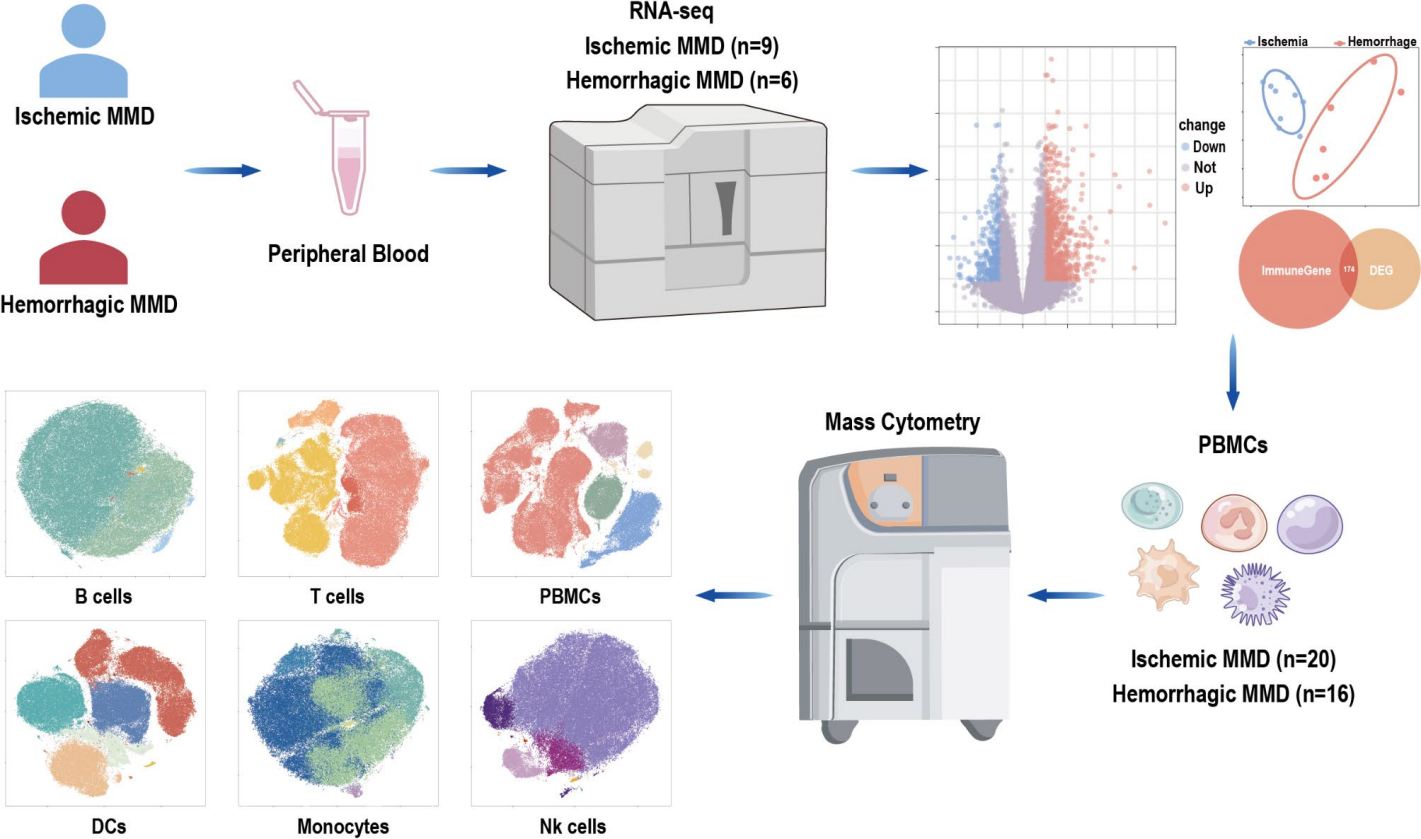
Experimental design. Comparing differences between ischemic and hemorrhagic moyamoya disease (MMD) patients using RNA sequencing and mass cytometry. PBMCs, peripheral blood mononuclear cells; NK, natural killer; DCs, dendritic cells

The RNA-seq raw matrix files (mRNA: HRA004479) were obtained from the China National Center for Bioinformation and are publicly available at https://ngdc.cncb.ac.cn/gsahuman. The initial CyTOF fcs files were obtained from OMIX, China National Center for Bioinformation (https://ngdc.cncb.ac.cn/omix: accession no. OMIX004669). The use and analysis of these data were authorized and approved by the corresponding authors [13]. The overall experimental design is presented in Fig. 1.

### RNA-seq data preprocessing

Prior to comprehensive analysis, we used the Data Table package for data cleaning and extraction. Genes with a maximum expression value below 10 across all samples were filtered out. For genes with duplicate names, the one with the highest expression was retained. Genes expressed in at least 50% of the samples were kept, ensuring that only genes consistently expressed in the majority of samples were included in subsequent analyses. The counts per million (CPM) of the expression matrix were calculated using the edgeR package [17], and the resulting cpm matrix was normalized using the Linear Models for Microarray Data (limma) package [18].

### Principal component analysis and differentially expressed genes (DEGs) screening

We performed principal component analysis (PCA) on the normalized data to visualize the overall data patterns among samples. The ischemic MMD group was used as the control group. Differential gene expression analysis was performed on the raw count matrix using the DESeq2 package [19]. The screening criteria for DEGs were set as an absolute log2 fold change > 1 and an adjusted *p-*value < 0.05.

### Functional enrichment analysis of DEGs

Gene Ontology (GO) classifies gene functions into biological process (BP), cellular component (CC), and molecular function (MF). The Kyoto Encyclopedia of Genes and Genomes (KEGG) helps understand biological systems via molecular data, with pathway enrichment analysis identifying key pathways in a gene set. We used the clusterProfiler package to perform GO and KEGG analyses on upregulated and downregulated genes to explore their biological functions and pathways [20].

### Gene set enrichment analysis (GSEA)

We performed GSEA with the clusterProfiler package to explore functional annotation differences between the two groups. The gene sets used included the hallmark gene sets and the ImmuneSigDB subset of C7 (immunologic signature gene sets). An adjusted *p*-value < 0.05 was used as a threshold to ensure the significance of the results in each analysis [21].

### Construction of Immune-Related gene set and Protein-Protein interaction network

Immune-related genes were obtained from ImmPort Portal (https://www.immport.org/home) and InnateDB: Systems Biology of the Innate Immune Response (https://www.innatedb.com/index.jsp) [22, 23]. After removing duplicates, the final immune-related gene set was compiled. The intersection between the immune-related gene set and DEGs was identified. Protein-protein interaction (PPI) networks were constructed and analyzed using the STRING network tool (https://string-db.org/) with a parameter setting of 0.9 to select high-confidence interactions [24]. The identified genes were considered immune-related hub genes, and the PPI network results were visualized using Cytoscape software [25].

### CyTOF data processing

For each sample, a manual gating strategy was used, and data from individual live cells were exported from the cell repository after preprocessing. Arcsinh normalization was then applied to identify clustering patterns accurately. High-dimensional data were analyzed using the PhenoGraph algorithm and the R package ‘cytofkit (v1.10.0)’. High-dimensional data were visualized in two dimensions using the t-Distributed Stochastic Neighbor Embedding (t-SNE) algorithm to elucidate clustering frequencies, cluster heterogeneity, and marker expression differences between the two groups. Clusters with similar phenotypic characteristics were merged manually. The proportions of cell clusters and marker expression levels were extracted and analyzed statistically using the R packages ‘pheatmap (v1.0.12)’ and ‘ggplot2 (v3.4.0)’ [26].

### Gating strategy

The analysis of circulating CD45^+^ immune cells followed a multi-step gating process. Initially, five major clusters were identified using specific markers: T cells (CD3), B cells (CD19), natural killer (NK) cells (CD56), monocytes (CD14), and dendritic cells (CD3^−^ CD19^−^ CD56^−^ CD14^−^). The corresponding cell populations were gated using FlowJo software, followed by dimensionality reduction and visualization of the high-dimensional data. This gating strategy enabled accurate identification and analysis of the distribution and expression profiles of different immune cell types in the samples [27].

### Statistical analysis

Statistical analyses was conducted using SPSS (version 26.0). Continuous variables were expressed as mean ± standard deviation (SD), and categorical variables as frequencies. Comparisons between the two groups were made using the Wilcoxon test for continuous variables and the χ² test for categorical variables. The Wilcoxon rank-sum test was used to compare cell cluster proportions and marker expression levels. A two-sided p-value of less than 0.05 was considered statistically significant.

## Results

### Transcriptome differences in peripheral blood of ischemic and hemorrhagic MMD patients

**Fig. 2:**
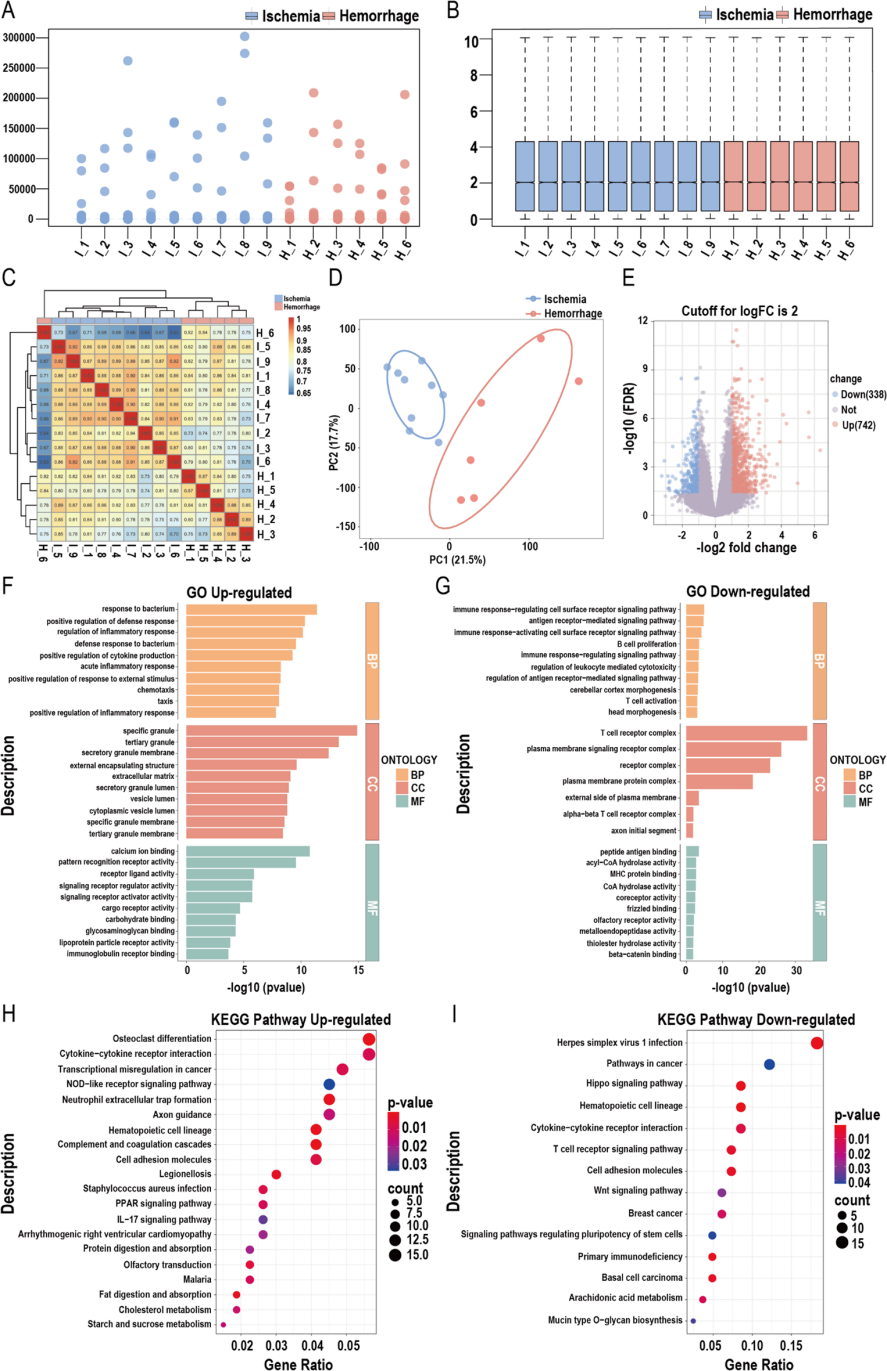
Analysis of peripheral blood transcriptome in ischemic and hemorrhagic MMD patients. (**A**) Distribution of raw, unprocessed data. (**B**) Distribution of processed data. (**C**) Heatmap of sample correlations. (**D**) Principal component analysis (PCA) showing relationships between samples. (**E**) Volcano plot of differentially expressed genes. Compared to ischemic group, (**F**) Gene Ontology (GO) analysis of upregulated genes; (**G**) GO analysis of downregulated genes; (**H**) Kyoto Encyclopedia of Genes and Genomes (KEGG) analysis of upregulated genes; (**I**) KEGG analysis of downregulated genes

A total of 9 patients with ischemic MMD and 6 with hemorrhagic MMD were included in the study, based on baseline matching (Supplementary Table 1). Figure 2A shows the distribution of the unprocessed transcriptome data, while Fig. 2B presents the normalized data after processing. The heatmap of sample correlations after processing is shown in Fig. 2C. Figure 2D illustrates the PCA plot after dimensionality reduction, demonstrating good clustering within each group. The volcano plot (Fig. 2E) indicates that, compared to the ischemic group, 742 genes were upregulated and 338 genes were downregulated in the hemorrhagic group. GO enrichment analysis of DEGs between the ischemic and hemorrhagic groups (Fig. 2F-G) showed that patients with hemorrhagic MMD exhibited an immune activation state, while the functions of immune cells and receptor signaling pathways were suppressed. KEGG analysis (Fig. 2H-I) further revealed that, compared to patients with ischemic MMD, those with hemorrhagic MMD exhibited immune dysregulation and dysfunction of immune cells.

### GSEA and PPI network

**Fig. 3:**
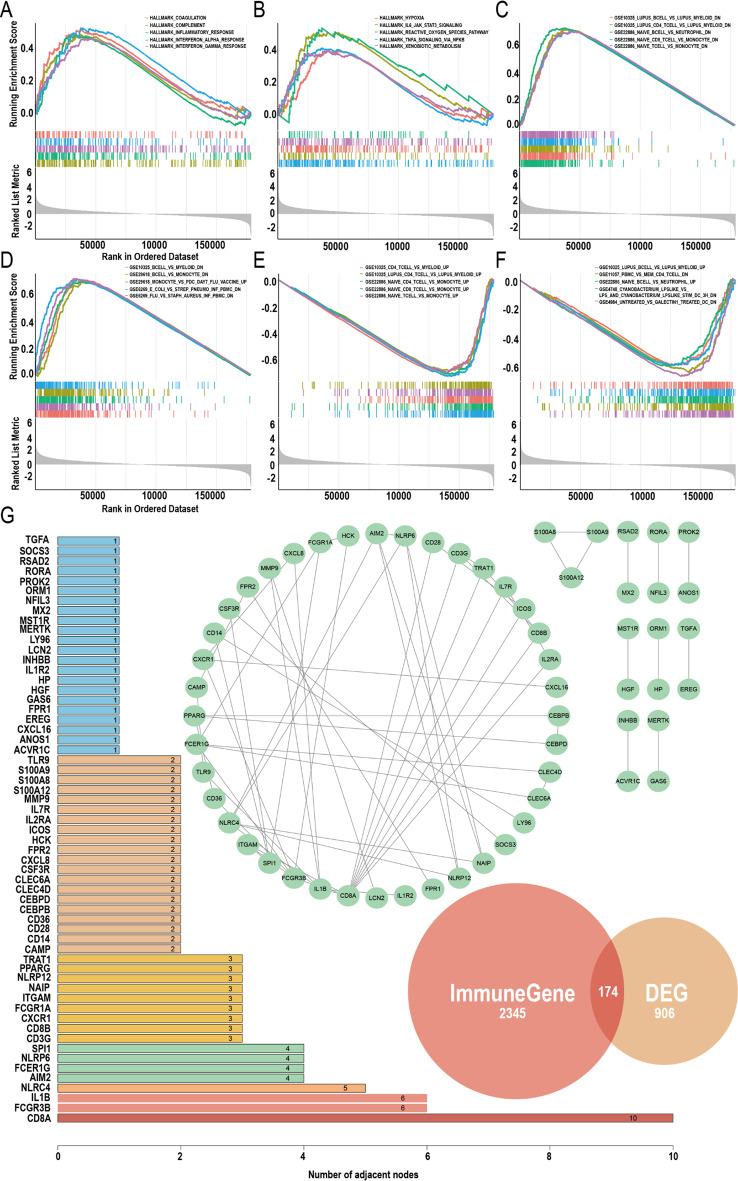
Correlation analysis between peripheral blood transcriptome and immunity. (**A**-**B**) Top 10 upregulated pathways from Gene Set Enrichment Analysis (GSEA) of hallmark gene sets (adjust *p* < 0.05). (**C**-**D**) Top 10 upregulated pathways from GSEA of ImmuneSigDB C7 subset (immunologic signature gene sets, adjust *p* < 0.05). (**E**-**F**) Top 10 downregulated pathways from GSEA analysis of ImmuneSigDB subset of C7 (adjust *p* < 0.05). (**G**) Protein-protein interaction (PPI) network of the intersection between immune gene sets and differentially expressed genes and the node count of key genes

GSEA identified several upregulated pathways in the hemorrhagic group across the Hallmark gene set. The pathways that were enriched included coagulation, complement, inflammatory response, hypoxia, IL-6/JAK/STAT3 signaling, reactive oxygen species pathway, and TNF-α signaling via NF-κB (Fig. 3A and B). These findings indicate that the hemorrhagic phenotype in MMD is associated with enhanced activity in pathways related to coagulation, immune response, hypoxia, and inflammation. For the ImmuneSigDB subset of C7, GSEA analysis (Fig. 3C–F) revealed that the differences between ischemic and hemorrhagic MMD were primarily associated with the imbalance between adaptive immune cells (T and B cells) and innate immune cells (myeloid, monocytes, neutrophils). Additionally, the intersection of DEGs and immune-related genes resulted in 174 genes. Among these, 62 core genes demonstrated high-confidence interactions with a confidence score of 0.9 in the PPI network. The number of connections within the PPI network and the core genes is shown in Fig. 3G, and the core genes are listed in Supplementary Table 2.

### Higher proportion of NK cells and lower proportion of monocytes in hemorrhagic MMD patients

**Fig. 4:**
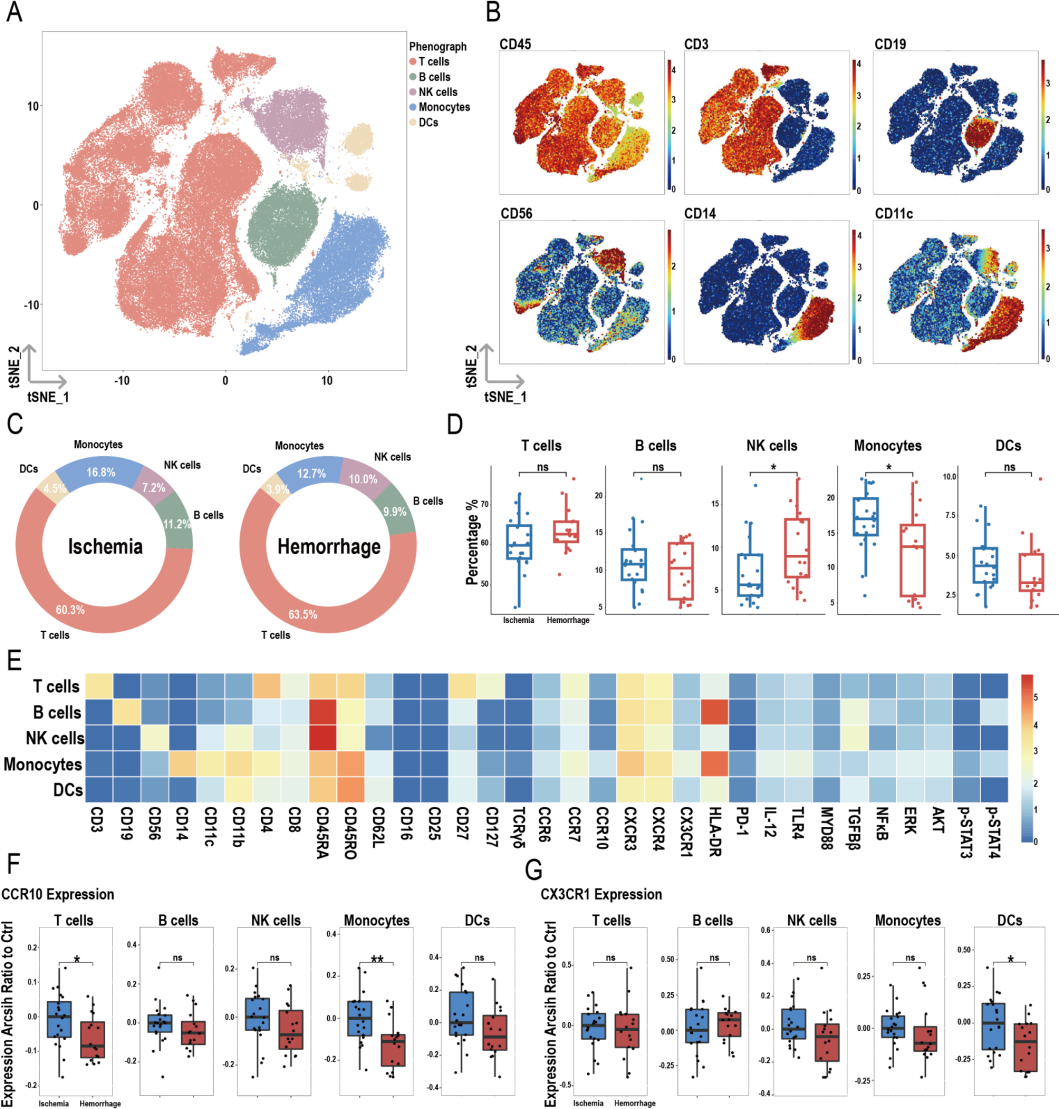
Overview of imaging mass cytometry on PBMCs in ischemic and hemorrhagic MMD patients. (**A**) Representative t-Distributed Stochastic Neighbor Embedding (t-SNE) plots of five PBMC clusters, including T cells, B cells, NK cells, monocytes, and DCs from MMD patients. (**B**) t-SNE heatmap of cluster markers. (**C**) Donut chart showing the proportions of PBMC subsets in ischemic and hemorrhagic MMD patients. (**D**) Bar chart comparing PBMC subset proportions between ischemic and hemorrhagic groups. (**E**) Heatmap showing protein expression levels across PBMC clusters. (**F**) Expression differences of CCR10 between ischemic and hemorrhagic groups in PBMC clusters. (**G**) Expression differences of CX3CR1 between ischemic and hemorrhagic groups in PBMC clusters. Significance: ns, *p* ≥ 0.05; *p < 0.05; **p < 0.01

Twenty ischemic MMD patients and sixteen hemorrhagic MMD patients were included in the study after baseline matching (Supplementary Table 3). The mass cytometry antibody panel is provided in Supplementary Table 4. After dimensionality reduction and clustering of CD45^+^ immune cells, five subpopulations were identified: T cells, B cells, NK cells, monocytes, and dendritic cells (DCs) (Fig. 4A). Their respective markers are shown in Fig. 4B, with more detailed subpopulation information provided in Table 1. Figure 4C shows the proportions of these five subpopulations in the ischemic and hemorrhagic groups. Compared to the ischemic group, the hemorrhagic group had a higher proportion of NK cells and a lower proportion of monocytes (Fig. 4D). Figure 4E displays the heatmap of various molecules within the five subpopulations. Expression of CCR10 was lower in T cells and monocytes of hemorrhagic MMD patients (Fig. 4F). For CX3CR1, expression was decreased only in DCs in the hemorrhagic group compared to the ischemic group (Fig. 4G). No significant differences were observed in the expression of other molecules between the two groups within the five subpopulations (Figure S1A-E).

**Table 1:** Comparison between phenograph clusters and manually gated cell phenotypes

Cell Lineages	PhenoGraph Clusters	Cell Phenotypes
T cells	T cells	CD3^+^
	CD4^+^T cells	CD4^+^
	CD8^+^T cells	CD8^+^
	DPT cells	CD4^+^CD8^+^
	DNT cells	CD4^−^CD8^−^
	NKT cells	CD56^+^
	Treg cells	CD4^+^CD25^+^
	γδT cells	TCRγδ^+^
B cells	B cells	CD19^+^
	B01	CD45RA^low^ CD27^−^
	B02	CD27^+^
	B03	CD27^high^
	B04	CCR7^+^
	B05	PD-1^+^
NK cells	NK cells	CD56^+^
	NK01	CD45RA^high^ HLA-DR^−^
	NK02	CD56^high^
	NK03	CD45RA^high^ HLA-DR^+^
	NK04	HLA-DR^high^
	NK05	PD-1^+^
	NK06	CCR7^high^
	NK07	CXCR4^high^
Monocytes	Monocytes	CD14^+^
	M01	CD45RO^high^
	M02	CD45RO^low^ CD45RA^high^
	M03	HLA-DR^high^
	M04	CD45RO^low^ CD45RA^low^ HLA-DR^low^
	M05	PD-1^+^
	M06	CCR7^+^
DCs	DCs	CD3^−^CD19^−^CD56^−^CD14^−^
	DC01	HLA-DR^high^
	DC02	CD11b^+^CD45RA^high^ CD45RO^high^
	DC03	CD11b^+^CD45RA^high^ CD45RO^low^
	DC04	CD11b^+^CD45RA^low^ CD45RO^high^
	DC05	CD11b^−^CCR6^−^CXCR4^−^CD45RA^low^
	DC06	CXCR4^high^
	DC07	CCR6^+^

DCs indicate dendritic cells; DNT, double-negative T; DPT, double-positive T; and NK, natural killer

### Decreased CD3 expression and reduced chemotaxis of DPT in hemorrhagic MMD patients

**Fig. 5:**
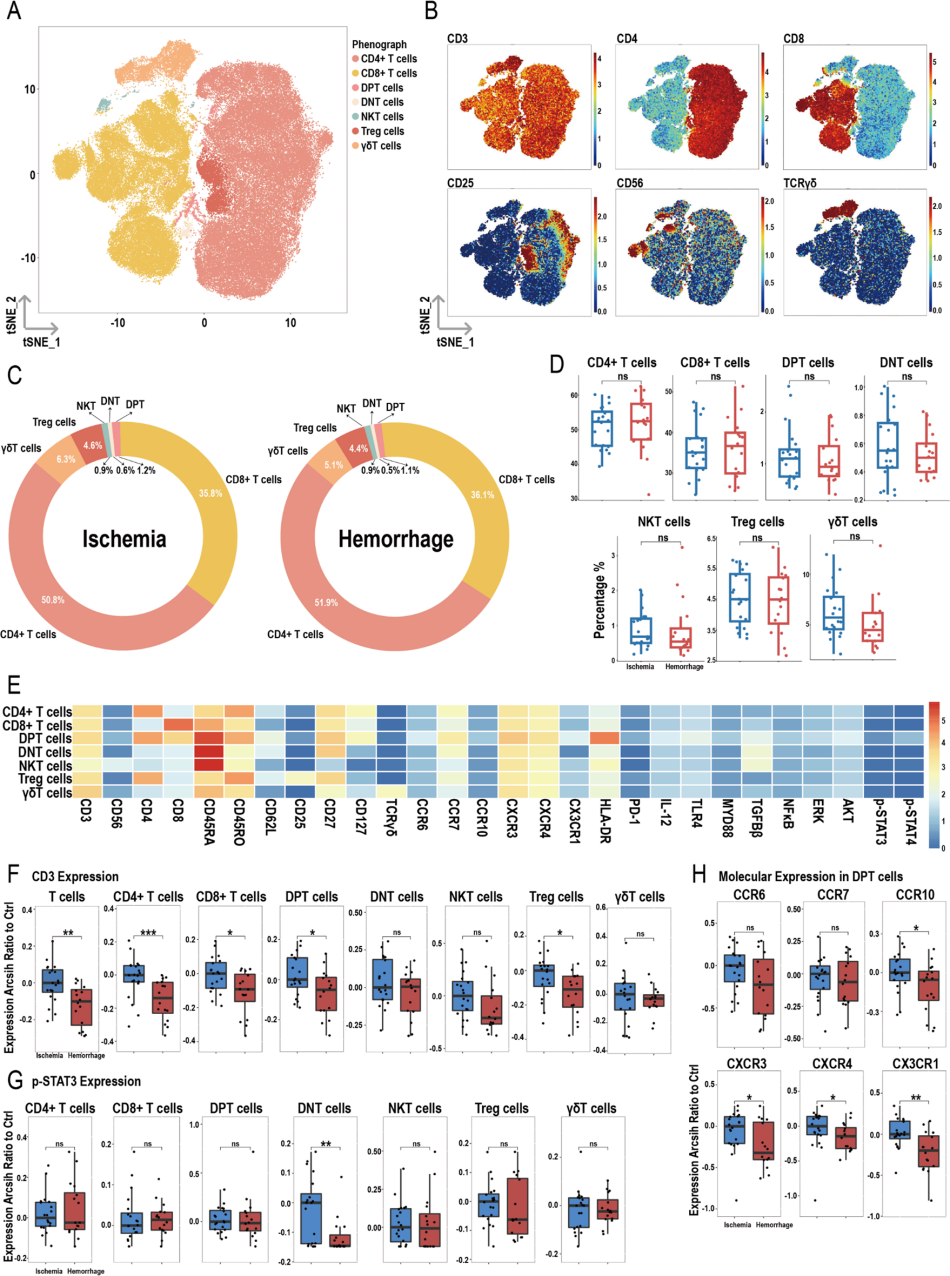
Overview of imaging mass cytometry on T cells in ischemic and hemorrhagic MMD patients. (**A**) Representative t-SNE plot of seven T cell clusters from MMD patients. (**B**) t-SNE heatmap of cluster markers. (**C**) Donut chart showing T cell subset proportions in ischemic and hemorrhagic MMD patients. (**D**) Bar chart comparing T cell subset proportions between ischemic and hemorrhagic groups. (**E**) Heatmap showing protein expression levels across T cell clusters. (**F**) Expression differences of CD3 between ischemic and hemorrhagic groups in T cells and corresponding clusters. (**G**) Expression differences of p-STAT3 between ischemic and hemorrhagic groups in T cell clusters. (**H**) Molecular expression in CD4^+^CD8^+^ double-positive T (DPT) cells. Significance: ns, *p* ≥ 0.05; *p < 0.05; **p < 0.01; ***p < 0.001

After dimensionality reduction and clustering of total T cells, seven subpopulations were identified: CD4^+^ T, CD8^+^ T, CD4^+^CD8^+^ double-positive T (DPT), CD4^−^CD8^−^ double-negative T (DNT), NKT, Treg, and γδT cells (Fig. 5A), with their respective markers shown in Fig. 5B. Figure 5C shows the proportions of these seven subpopulations in the ischemic and hemorrhagic groups, with no significant differences in the proportions between the two groups (Fig. 5D). Figure 5E displays the heatmap of various molecules within the seven subpopulations.

CD3 expression was lower in the hemorrhagic group compared to the ischemic group in total T, CD4^+^ T, CD8^+^ T, DPT, and Treg cells (Fig. 5F). For p-STAT3, expression was decreased only in DNT cells in the hemorrhagic group (Fig. 5G). In DPT cells, the expressions of CCR10, CXCR3, CXCR4, and CX3CR1 were lower in the hemorrhagic group, while no differences were observed for CCR6 and CCR7 (Fig. 5H). Additionally, CD25 expression in Treg cells and TCRγδ expression in γδT cells were lower in the hemorrhagic group compared to the ischemic group. No significant differences were observed in the expression of other molecules within the T cell subpopulations between the two groups (Figure S2A-G).

### Differences in B cells between ischemic and hemorrhagic groups: focus on the B03 subpopulation

**Fig. 6:**
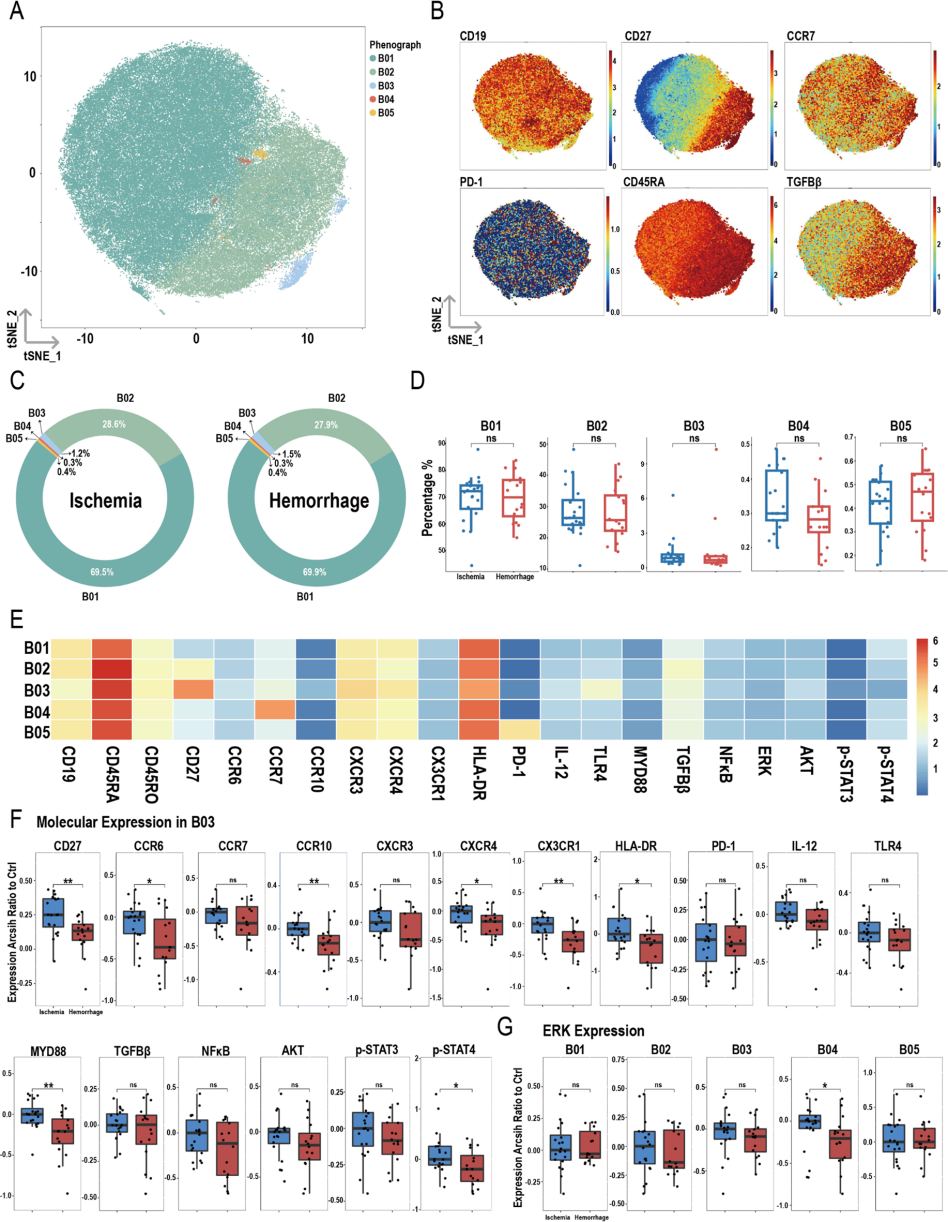
Overview of imaging mass cytometry on B cells in ischemic and hemorrhagic MMD patients. (**A**) Representative t-SNE plot of five B cell clusters from MMD patients. (**B**) t-SNE heatmap of cluster markers. (**C**) Donut chart showing B cell subset proportions in ischemic and hemorrhagic MMD patients. (**D**) Bar chart comparing B cell subset proportions between ischemic and hemorrhagic groups. (**E**) Heatmap showing protein expression levels across B cell clusters. (**F**) Molecular expression in B03 cluster. (**G**) Expression differences of ERK between ischemic and hemorrhagic groups in B cell clusters. Significance: ns, *p* ≥ 0.05; *p < 0.05; **p < 0.01

After dimensionality reduction and clustering of total B cells, five subpopulations were identified: B01, B02, B03, B04, and B05 (Fig. 6A), with their respective markers shown in Fig. 6B. Figure 6C presents the proportions of these five subpopulations in the ischemic and hemorrhagic groups, with no significant differences between the two groups (Fig. 6D). Figure 6E displays the heatmap of various molecules within the subpopulations.

In the B03 cluster, expression levels of CD27, CCR6, CCR10, CXCR4, CX3CR1, HLA-DR, MYD88, and p-STAT4 were lower in the hemorrhagic group compared to the ischemic group, while other molecules showed no significant differences (Fig. 6F). For ERK, its expression was decreased only in the B04 cluster in the hemorrhagic group (Fig. 6G). No significant differences were observed in the expression of other molecules in the B01, B02, B03, and B05 subpopulations between the two groups (Figure S3A-D).

### The proportion of NK07 subpopulation is lower in hemorrhagic MMD patients

**Fig. 7:**
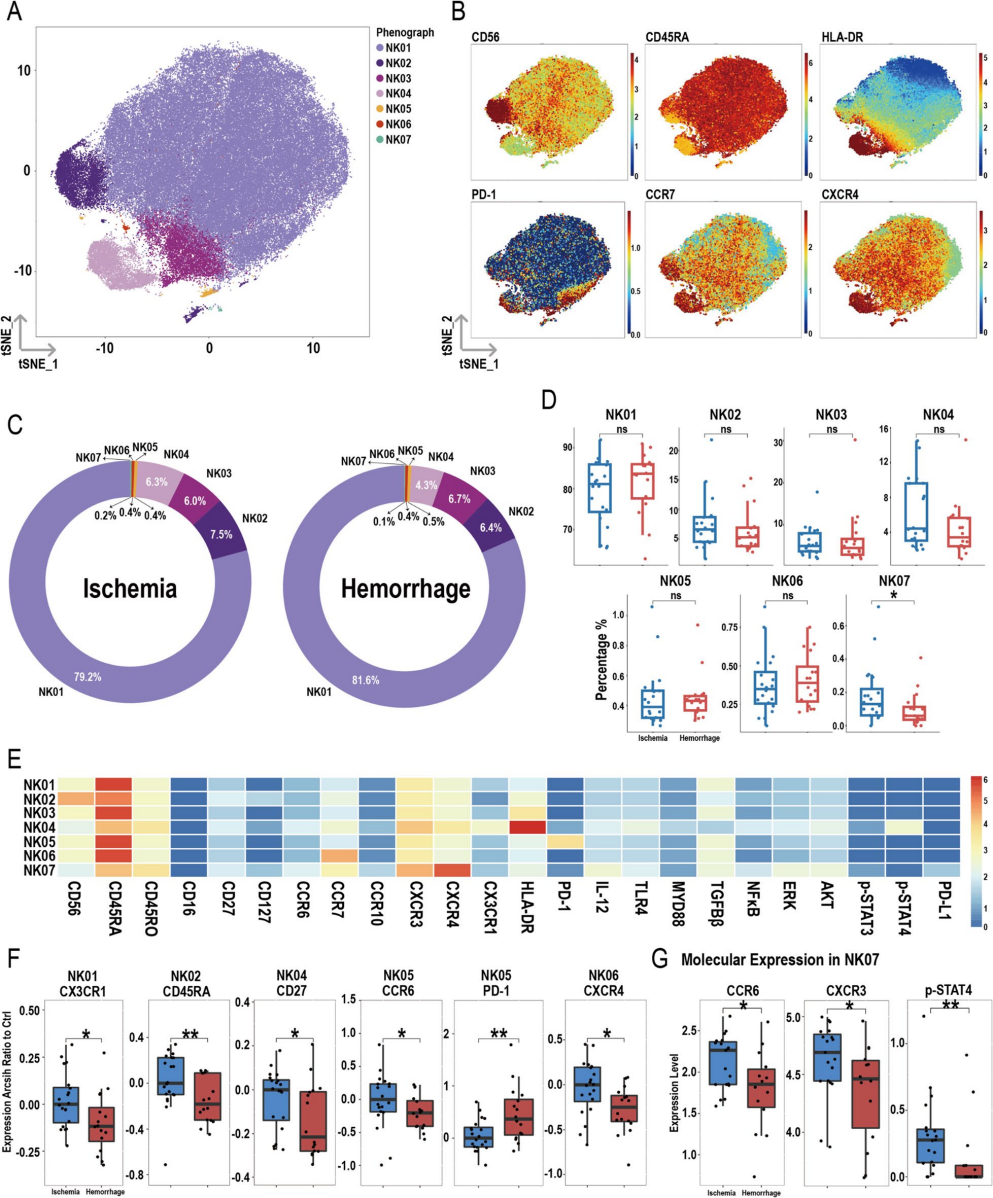
Overview of imaging mass cytometry on NK cells in ischemic and hemorrhagic MMD patients. (**A**) Representative t-SNE plot of seven NK cell clusters from MMD patients. (**B**) t-SNE heatmap of cluster markers. (**C**) Donut chart showing NK cell subset proportions in ischemic and hemorrhagic MMD patients. (**D**) Bar chart comparing NK cell subset proportions between ischemic and hemorrhagic groups. (**E**) Heatmap showing protein expression levels across NK cell clusters. (**F**) Differential molecular expression between ischemic and hemorrhagic groups in NK cell subsets. (**G**) Molecular expression in NK07 cluster. Significance: ns, *p* ≥ 0.05; *p < 0.05; **p < 0.01

After dimensionality reduction and clustering of total NK cells, seven subpopulations were identified: NK01, NK02, NK03, NK04, NK05, NK06, and NK07 (Fig. 7A), with their respective markers shown in Fig. 7B. Figure 7C presents the proportions of these subpopulations in the ischemic and hemorrhagic groups. The results indicate that the proportion of the NK07 cluster is lower in hemorrhagic MMD patients compared to the ischemic group, while the proportions of the other subpopulations showed no significant differences (Fig. 7D). Figure 7E displays the heatmap of various molecules within the subpopulations.

In the hemorrhagic group, the expression of CX3CR1 in NK01, CD45RA in NK02, CD27 in NK04, CCR6 in NK05, and CXCR4 in NK06 was lower compared to the ischemic group, while PD-1 expression in the NK05 cluster was higher (Fig. 7F). No significant differences were observed in the expression of other molecules within the NK01, NK02, NK03, NK04, NK05, and NK06 subpopulations (Figure S4A-F). In the NK07 subpopulation, the expressions of CCR6, CXCR3, and p-STAT4 were lower in the hemorrhagic group compared to the ischemic group (Fig. 7G), while other molecules showed no significant differences in the NK07 cluster (Figure S5).

### Reduced CCR10 in M02 and M04 clusters of hemorrhagic MMMD patients

**Fig. 8:**
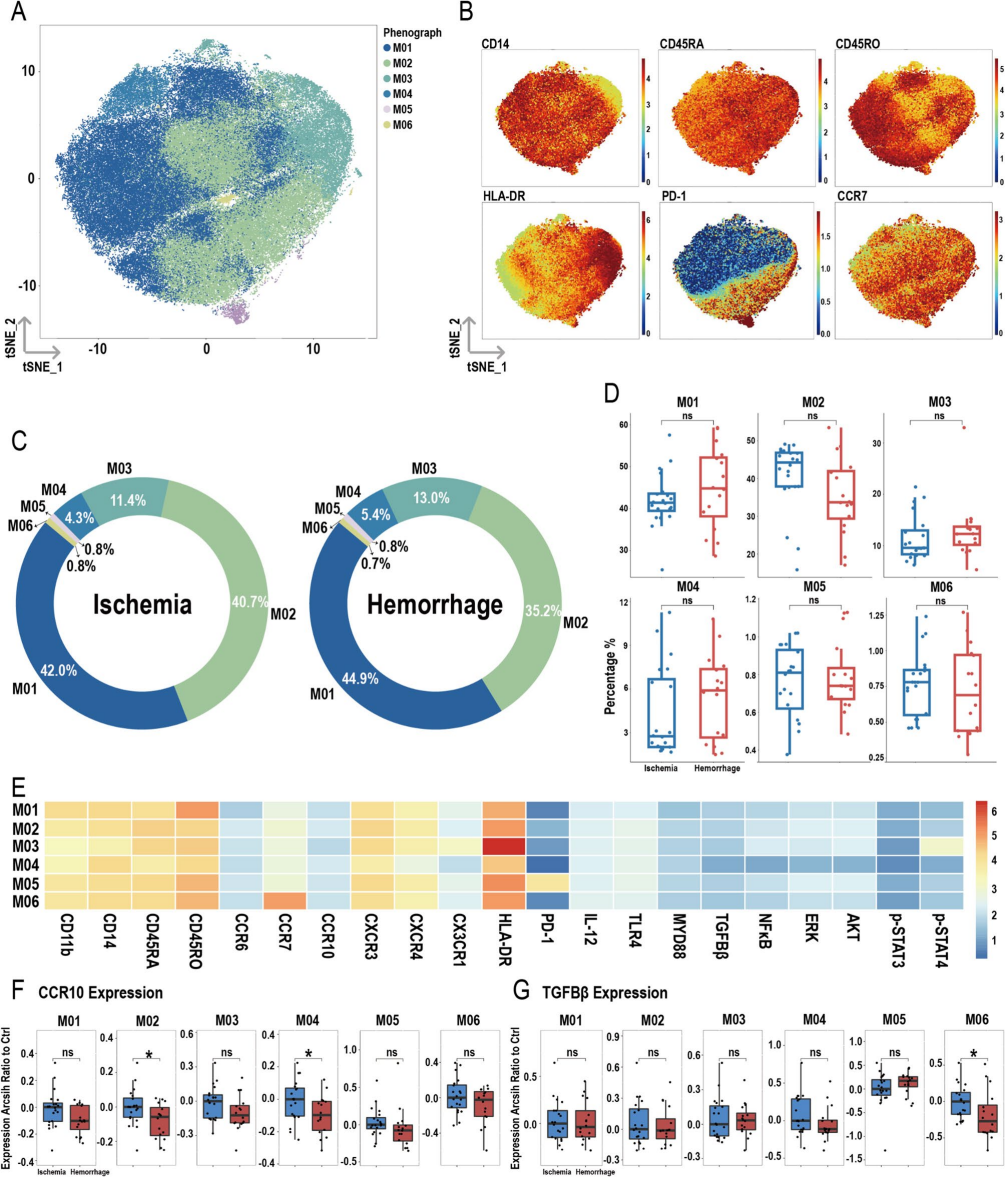
Overview of imaging mass cytometry on monocytes in ischemic and hemorrhagic MMD patients. (**A**) Representative t-SNE plot of six monocyte clusters from MMD patients. (**B**) t-SNE heatmap of cluster markers. (**C**) Donut chart showing monocyte subset proportions in ischemic and hemorrhagic MMD patients. (**D**) Bar chart comparing monocyte subset proportions between ischemic and hemorrhagic groups. (**E**) Heatmap showing protein expression levels across monocyte clusters. (**F**) Expression differences of CCR10 between ischemic and hemorrhagic groups in monocyte clusters. (**G**) Expression differences of TGFβ between ischemic and hemorrhagic groups in monocyte clusters. Significance: ns, *p* ≥ 0.05; *p < 0.05

After dimensionality reduction and clustering of the total monocytes, six subpopulations were identified: M01, M02, M03, M04, M05, and M06 (Fig. 8A), with their respective markers shown in Fig. 8B. Figure 8C presents the proportions of these subpopulations in the ischemic and hemorrhagic groups, with no significant differences in the proportions between the two groups (Fig. 8D). Figure 8E displays the heatmap of various molecules within the subpopulations.

In the M02 and M04 clusters, the expression of CCR10 was lower in the hemorrhagic group compared to the ischemic group (Fig. 8F). For TGF-β, its expression was decreased only in the M06 cluster in the hemorrhagic group (Fig. 8G). No significant differences were observed in the expression of other molecules within the M01, M02, M03, M04, and M05 subpopulations (Figure S6A-E). In the M06 subpopulation, the expression of CD11b was lower in the hemorrhagic group compared to the ischemic group, while other molecules showed no significant differences in the M06 subpopulation (Figure S6F).

### Increased proportions of D03 and D07 subpopulations in hemorrhagic MMD patients

**Fig. 9:**
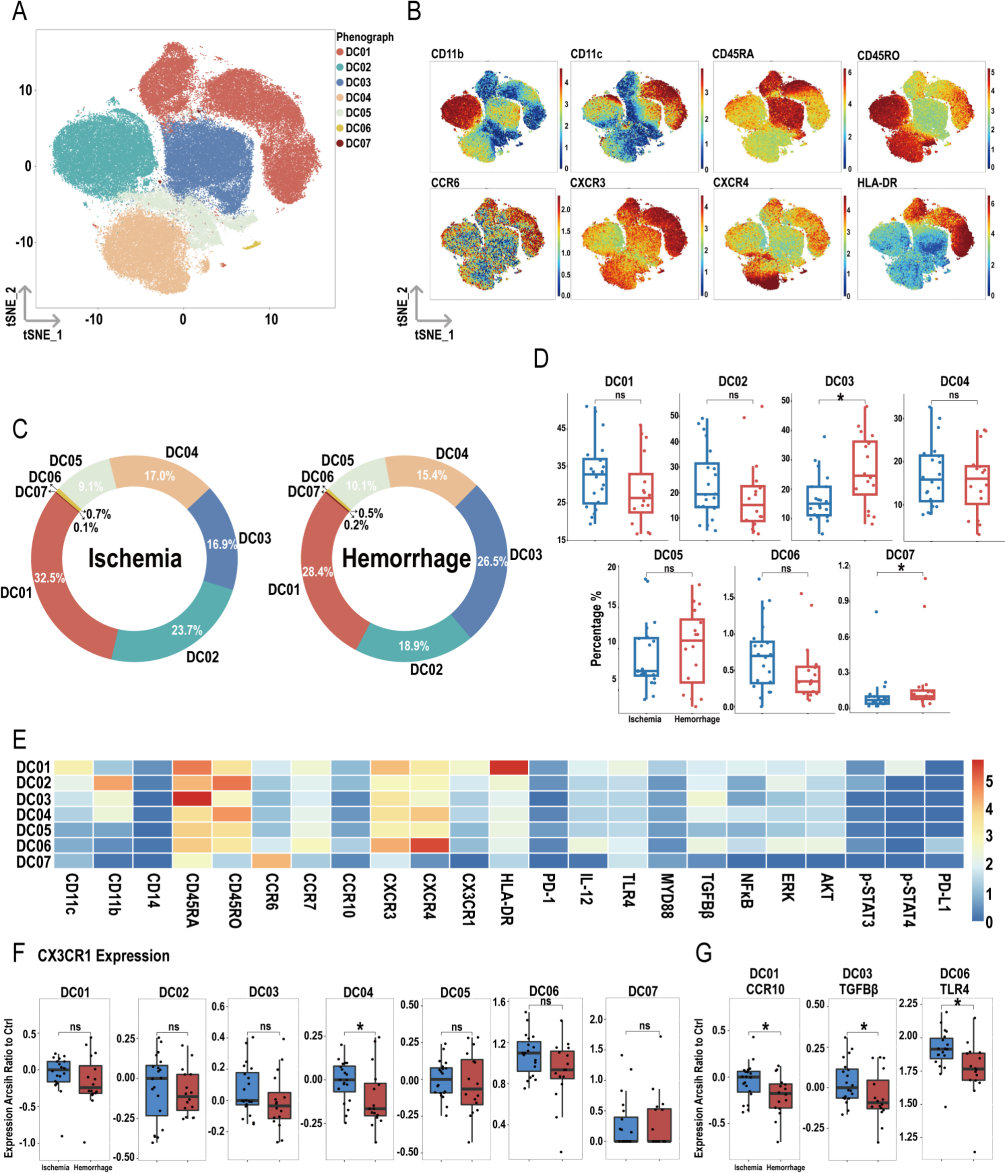
Overview of imaging mass cytometry on DCs in ischemic and hemorrhagic MMD patients. (**A**) Representative t-SNE plot of seven DC clusters from MMD patients. (**B**) t-SNE heatmap of cluster markers. (**C**) Donut chart showing DC subset proportions in ischemic and hemorrhagic MMD patients. (**D**) Bar chart comparing DC subset proportions between ischemic and hemorrhagic groups. (**E**) Heatmap showing protein expression levels across DC clusters. (**F**) Expression differences of CX3CR1 between ischemic and hemorrhagic groups in DC clusters. (**G**) Differential molecular expression between ischemic and hemorrhagic groups in DC01, DC03, DC06 clusters. Significance: ns, *p* ≥ 0.05; *p < 0.05

After dimensionality reduction and clustering of total DCs, seven subpopulations were identified: DC01, DC02, DC03, DC04, DC05, DC06, and DC07 (Fig. 9A), with their respective markers shown in Fig. 9B. Figure 9C presents the proportions of these subpopulations in the ischemic and hemorrhagic groups. The results indicate that the proportions of D03 and D07 subpopulations are higher in the hemorrhagic group compared to the ischemic group, while the other subpopulations showed no significant differences (Fig. 9D). Figure 9E displays the heatmap of various molecules within the subpopulations.

In the hemorrhagic group, the expression of CX3CR1 was lower only in the DC04 subpopulation compared to the ischemic group (Fig. 9F). Additionally, the expression of CCR10 in DC01, TGF-β in DC03, and TLR4 in DC05 was all lower in the hemorrhagic group (Fig. 9G). For the DC01 subpopulation, the expression of CD14 was higher in the hemorrhagic group compared to the ischemic group, whereas in the DC04 subpopulation, the expression of CD45RO was higher in the hemorrhagic group. No significant differences were observed in the expression of other molecules within the DC subpopulations (Figures S7-S8).

## Discussion

MMD is increasingly recognized as being significantly influenced by chronic inflammation [28–30]. Circulating immune cells play a crucial role in promoting MMD progression by infiltrating vessel walls and triggering pro-inflammatory molecules [31, 32]. However, research on circulating immunity in different MMD subtypes remains limited. In this study, we compared peripheral immune profiles between ischemic and hemorrhagic MMD patients. The hemorrhagic group exhibited upregulated genes associated with inflammation, hypoxia, and bacterial responses and downregulated genes related to immune response regulation. Mass cytometry analysis revealed reduced CD3 expression in T cells and their specific subsets, a higher proportion of NK cells, a lower proportion of monocytes, diminished expression of various molecules in B and NK cell subsets, and altered DC subsets in the hemorrhagic group.

T cell receptors (TCRs) are unique surface markers on T cells that regulate T cell function by recognizing and binding to exogenous antigens, participating in the body’s immune response to prevent immune dysregulation and inflammation [33, 34]. Due to their short intracellular segments, TCRs require intracellular molecules for signal transduction, with CD3 molecules being one of the most important co-molecules [35]. The TCR-CD3 complex is involved in crucial immunophysiological processes, including T cell development, antigen recognition, signal transduction regulation, and the activation of adaptive immune functions. Abnormal expression of CD3 can induce various immune system diseases [36]. Research on Alzheimer’s disease suggests that a decrease in the level of CD3 lymphocytes in peripheral blood can trigger systemic immune and inflammatory changes, including mitochondrial dysfunction, increased oxidative stress, and reduced protein clearance [37]. Although there is no significant difference in the overall proportions of T cells and their subsets between ischemic and hemorrhagic groups, the expression of CD3 in total T cells, CD4^+^ T cells, CD8^+^ T cells, DPT cells, and Treg cells subsets is decreased in hemorrhagic MMD patients during the non-acute phase. This finding aligns with transcriptome data showing decreased CD3G expression in the hemorrhagic group, along with downregulated enriched pathways (T cell activation, TCR complex, TCR signaling pathway). These results suggest that T cells and their specific subsets in hemorrhagic MMD may undergo some degree of functional suppression, potentially leading to a weakened or dysregulated immune response.

DPT cells, an unconventional mature T cell population, represent a subset with a unique maturation phenotype and are differentiated effector memory cells with antiviral functions [38–40]. Studies have shown an increased proportion of DPT cells in patients with viral infections and various autoimmune diseases, suggesting that this small T cell population may play a role in virus clearance. In some cases, the DPT cell population can serve as a marker for assessing disease severity and progression [41]. Compared to ischemic MMD, hemorrhagic MMD patients show no difference in the number of DPT cells, but their expression of chemokine receptors CCR10, CXCR3, CXCR4, and CX3CR1 is reduced, indicating impaired chemotactic ability. This suggests that the ability of DPT cells to migrate to relevant sites and exert antiviral functions is diminished in hemorrhagic MMD patients. Additionally, we observed a decrease in the expression of the chemokine receptor CCR10 in the entire T cell population of hemorrhagic MMD patients, further indicating a weakened overall chemotactic ability of T cells.

The efficiency of immune responses triggered by immunological memory ensures rapid clearance of the infection source upon re-exposure. This memory is mediated by memory T cells, B cells, and plasma cells [42, 43]. In our study, we identified two B cell subsets, B02 and B03, both expressing CD27, a marker commonly used to define human memory B cells [44]. Among all B cell subsets, plasma cells exhibit the highest levels of CD27 expression, although mature plasma cells lose CD19 expression [45, 46]. Therefore, although the B03 subset shows the highest CD27 expression, its CD19 expression suggests that B03 is a memory B cell subset with higher CD27 expression compared to B02. Memory B cells are the primary precursor cells for new plasma cells during secondary infection. Compared to ischemic MMD, we found that the expression of several molecules in the B03 subset (CD27^high^) was significantly reduced in the hemorrhagic group. These molecules include CD27, CCR6, CCR10, CXCR4, CX3CR1, HLA-DR, MYD88, and p-STAT4. This suggests that the antigen presentation, T-cell stimulation, chemotactic ability, and immune response capacity of the B03 subset are decreased in patients with hemorrhagic MMD, indicating impaired immune memory in these patients.

Human NK cells constitute 15% of circulating lymphocytes and are involved in killing infected microbes and malignant autologous and allogeneic cells [47]. Through innate cytotoxicity, cytokine and chemokine production, and migration capabilities, NK cells play a crucial immunoregulatory role in the initiation and chronicization of inflammatory and autoimmune responses [48]. An increase in NK cells is observed in viral infections and early-stage tumor patients (when there is still an antitumor immune response) [49, 50]. In the synovial fluid of patients with rheumatoid arthritis, the accumulation of NK cells contributes to joint inflammation [51]. In our study, we found an increased proportion of NK cells in hemorrhagic MMD patients, consistent with the enrichment of upregulated genes in the hemorrhagic group in transcriptomic data. This suggests that the infectious burden in hemorrhagic MMD may be heavier than in ischemic MMD and that chronic inflammation may lead to an increased proportion of NK cells. Despite the increased NK cell proportion in hemorrhagic MMD patients, the chemotactic ability of their subpopulations is reduced. We observed decreased expression of CX3CR1 in the NK01 subpopulation, CCR6 in the NK05 subpopulation, CXCR4 in the NK06 subpopulation, and both CCR6 and CXCR3 in the NK07 subpopulation. The function of the increased NK cells in hemorrhagic-type MMD requires further investigation in future studies.

Meanwhile, it was found that the proportion of NK07 (CXCR4^high^) in the hemorrhagic group was lower than in the ischemic group among NK cell subsets. The main ligand of CXCR4 is the chemokine CXCL12 [52]. Studies in mice have shown that the CXCL12-CXCR4 axis in the blood-brain barrier is crucial for NK cells to enter the lesion area in stroke. These NK cells can protect the brain and improve motor abilities of mice after stroke induction [53]. This suggests that NK07 may be a protective NK cell subset, and its reduced proportion in the hemorrhagic group could indicate a weakened protective effect of circulating immunity on the brain.

Monocytes play a crucial role in maintaining homeostasis, pathogen recognition and clearance, and inflammation processes [54]. In a mouse model study, researchers found that during the acute phase of severe inflammation, monocytes were significantly reduced due to apoptosis, and the remaining monocytes in the bone marrow exhibited dysfunction [55]. Our research revealed that patients with hemorrhagic MMD had a decreased proportion of monocytes, along with reduced expression of CCR10 in both total peripheral monocytes and their subsets M02 and M04. This suggests a diminished ability of these monocytes to migrate and aggregate at inflammatory sites, thereby reducing their pathogen clearance function. Additionally, in hemorrhagic MMD patients, there was a decrease in CCR10 expression in DC subset D01 (HLA-DR^high^). As antigen-presenting cells, DCs in hemorrhagic MMD showed decreased expression of CX3CR1 in total DCs and their D04 subset, while the proportions of D03 (CD11b^+^ CD45RA^high^ CD45RO^low^) and D07 (CCR6^+^) increased, possibly indicating different functions. Overall, the reduced chemotactic ability of DCs in hemorrhagic MMD may be linked to the hemorrhagic phenotype.

Our study comprehensively describe the peripheral immune profiles of patients with ischemic and hemorrhagic MMD; however, there are still several potential directions for future research. First, the sample size in this study is relatively small, and single-cell technologies were not employed for further validation. Second, the study primarily focused on the proportion and chemotaxis of circulating immune cells, while the molecular pathway differences between the subgroups of MMD have not been fully explored. Future research should focus on a deeper investigation of immune cell-associated molecular pathways and their interactions with vascular-related cells.

## Conclusions

This study reveals significant differences in peripheral immune profiles between ischemic and hemorrhagic MMD, emphasizing the necessity of developing tailored therapeutic strategies for different MMD subtypes, and providing new insights into the immune pathogenesis underlying the distinct MMD subtypes.

## Electronic supplementary material

Below is the link to the electronic supplementary material.

Supplementary Material 1

## Data Availability

The data analyzed in this paper are from the Genome Sequence Archive (mRNA: HRA004479) and OMIX (accession no. OMIX004669), both at the National Genomics Data Center, China National Center for Bioinformation/Beijing Institute of Genomics, Chinese Academy of Sciences. The raw sequence data are accessible at https://ngdc.cncb.ac.cn/gsa/human,, and the CyTOF data at https://ngdc.cncb.ac.cn/omix. The code and data generated during the study are available from the corresponding author upon reasonable request.

## Abbreviations

BP: Biological process
CC: Cellular component
CPM: Counts per million
DCs: Dendritic cells
DEGs: Differentially expressed genes
DSA: Digital subtraction angiography
DNT: Double-negative T
DPT: Double-positive T
EDAS: Encephalo-duro-arterio-synangiosis
GO: Gene Ontology
GSEA: Gene set enrichment analysis
KEGG: Kyoto Encyclopedia of Genes and Genomes
Limma: Linear models for microarray data
MF: Molecular function
MMD: Moyamoya disease
NK: Natural killer
PBMCs: Peripheral blood mononuclear cells
PCA: Principal component analysis
PPI: Protein-protein interaction
SD: Standard deviation
TCRs: T cell receptors
TIA: Transient ischemic attacks
t-SNE: t-Distributed Stochastic Neighbor Embedding
