# Supplementary material for: Integrated bulk RNA sequencing and mass cytometry analysis reveal the circulating immune landscape in ischemic and hemorrhagic Moyamoya disease
Source: BMC Immunol. 2025 Mar 10;26:19. doi: 10.1186/s12865-025-00699-3 (PMC11892264; doi:10.1186/s12865-025-00699-3)
Supplement: Supplementary file 1 — Supplementary Material 1 [file 12865_2025_699_MOESM1_ESM.docx]

| **Supplementary Table 1. Clinical Characteristics of Patients with Ischemic and Hemorrhagic MMD in RNA-seq Cohort.** | | | |
| --- | --- | --- | --- |
| Characteristics | Ischemic MMD (n=9) | Hemorrhagic MMD (n=6) | *p* value |
| Age, y, mean ± SD | 32.56 ± 8.22 | 33.00 ± 7.85 | 0.953 |
| Female/male ratio | 6/3 | 3/3 | 0.622 |
| Clinical features, mean ± SD |  |  |  |
| Heart rate, bpm | 80.89 ± 9.28 | 76.17 ± 5.60 | 0.374 |
| SBP, mmHg | 124.00 ± 15.38 | 127.17 ± 13.35 | 0.679 |
| DBP, mmHg | 80.78 ± 9.14 | 78.50 ± 7.15 | 0.592 |
| BMI, kg/m² | 23.62 ± 2.95 | 26.66 ± 5.44 | 0.346 |
| *RNF213* p.R4810K, n (%) |  |  | 0.622 |
| Wild-type | 3 (33.3) | 3 (50.0) |  |
| Mutant | 6 (66.7) | 3 (50.0) |  |
| Vascular risk factors |  |  |  |
| Hypertension | 3 (33.3) | 2 (33.3) | 1.000 |
| Hypercholesterolemia | 1 (11.1) | 1 (16.7) | 1.000 |
| Diabetes mellitus | 0 (0.0) | 0 (0.0) | - |
| Current cigarette smoking | 1 (11.1) | 1 (16.7) | 1.000 |
| Current alcohol | 1 (11.1) | 0 (0.0) | 1.000 |

Supplementary Material

MMD, moyamoya disease; SD, standard deviation; SBP, systolic blood pressure; DBP, diastolic blood pressure; and BMI, body mass index.

**Supplementary Table 2. Comparison of Key Immune Gene Alterations in Ischemic and Hemorrhagic MMD Patients Based on RNA-seq Data.**

| Gene Symbol | Log2 Fold Change* | *p-*value | Adjusted *p-*value | Regulated |
| --- | --- | --- | --- | --- |
| HP | 4.308034444 | 1.4256E-08 | 2.10412E-06 | Up |
| ANOS1 | 3.049735341 | 0.000268944 | 0.003238748 | Up |
| S100A12 | 2.515445135 | 3.79398E-05 | 0.000750115 | Up |
| EREG | 2.420092549 | 0.001632388 | 0.012295581 | Up |
| MMP9 | 2.076640473 | 2.84241E-05 | 0.000608663 | Up |
| SOCS3 | 1.976129648 | 0.000116028 | 0.001753511 | Up |
| INHBB | 1.960091889 | 0.000211525 | 0.002719675 | Up |
| FCGR1A | 1.95291898 | 5.23237E-09 | 9.5352E-07 | Up |
| ORM1 | 1.906314743 | 5.88851E-06 | 0.00019296 | Up |
| CLEC4D | 1.885533118 | 0.000589846 | 0.00570952 | Up |
| RSAD2 | 1.84693967 | 0.006771798 | 0.034973264 | Up |
| CLEC6A | 1.821554201 | 0.003533681 | 0.021942977 | Up |
| MST1R | 1.718919213 | 0.004168573 | 0.02465349 | Up |
| PROK2 | 1.694835922 | 0.000575302 | 0.005623604 | Up |
| PPARG | 1.632988549 | 0.006936573 | 0.035616644 | Up |
| TGFA | 1.613323807 | 0.000844166 | 0.007526688 | Up |
| S100A8 | 1.612296718 | 0.001961995 | 0.014151562 | Up |
| HGF | 1.58949622 | 2.17987E-06 | 9.26914E-05 | Up |
| S100A9 | 1.583413784 | 2.43171E-06 | 9.82006E-05 | Up |
| NLRC4 | 1.524251582 | 1.2489E-07 | 1.088E-05 | Up |
| CAMP | 1.471372977 | 1.74328E-07 | 1.41515E-05 | Up |
| LCN2 | 1.409426009 | 0.004045704 | 0.02415977 | Up |
| CD14 | 1.353752694 | 2.50582E-12 | 3.72929E-09 | Up |
| CD36 | 1.29822423 | 0.000103517 | 0.001602005 | Up |
| FCER1G | 1.277694277 | 1.75452E-07 | 1.41783E-05 | Up |
| AIM2 | 1.27574528 | 0.000326254 | 0.003692874 | Up |
| IL1B | 1.247949131 | 1.20771E-06 | 5.76659E-05 | Up |
| MERTK | 1.228357044 | 0.00305564 | 0.019714842 | Up |
| TLR9 | 1.220376239 | 0.000164566 | 0.002270934 | Up |
| ITGAM | 1.207878741 | 1.53439E-06 | 6.99048E-05 | Up |
| CEBPB | 1.194480151 | 5.44008E-06 | 0.000181258 | Up |
| GAS6 | 1.187685367 | 3.00983E-06 | 0.000115102 | Up |
| NAIP | 1.180789284 | 6.83757E-05 | 0.001166305 | Up |
| NLRP12 | 1.156481268 | 3.82922E-07 | 2.56128E-05 | Up |
| LY96 | 1.130483233 | 0.003441666 | 0.021468637 | Up |
| IL1R2 | 1.095102853 | 0.000974109 | 0.008367212 | Up |
| FCGR3B | 1.085724319 | 0.000306233 | 0.00354684 | Up |
| FPR1 | 1.07996267 | 1.69982E-07 | 1.39551E-05 | Up |
| CXCR1 | 1.077937943 | 4.40954E-07 | 2.84155E-05 | Up |
| HCK | 1.076887583 | 3.19146E-07 | 2.20063E-05 | Up |
| CSF3R | 1.063269385 | 2.69395E-10 | 1.02364E-07 | Up |
| NLRP6 | 1.050903849 | 2.55618E-05 | 0.000571349 | Up |
| SPI1 | 1.048649292 | 3.11311E-06 | 0.000118544 | Up |
| FPR2 | 1.026149103 | 2.08849E-05 | 0.000489481 | Up |
| CEBPD | 1.021881872 | 3.1836E-05 | 0.000661885 | Up |
| CXCL16 | 1.018765917 | 2.27005E-06 | 9.43523E-05 | Up |
| MX2 | 1.00446316 | 8.02954E-05 | 0.001327773 | Up |
| NFIL3 | 1.000159826 | 4.9901E-06 | 0.000168509 | Up |
| IL2RA | -1.008135298 | 0.000111697 | 0.001702052 | Down |
| CD8A | -1.016863461 | 8.89885E-05 | 0.001439534 | Down |
| CD8B | -1.032587734 | 2.2124E-05 | 0.000510482 | Down |
| CD3G | -1.096573452 | 9.81667E-08 | 9.26033E-06 | Down |
| IL7R | -1.17742267 | 1.46795E-06 | 6.75675E-05 | Down |
| RORA | -1.217850403 | 3.88666E-09 | 7.58591E-07 | Down |
| CD28 | -1.222738749 | 4.22423E-08 | 4.96319E-06 | Down |
| TRAT1 | -1.364773186 | 3.48236E-05 | 0.000706721 | Down |
| ICOS | -1.471726283 | 2.12227E-06 | 9.10745E-05 | Down |
| CXCL8 | -1.691063276 | 1.47233E-05 | 0.00037403 | Down |
| ACVR1C | -1.889903658 | 0.001599022 | 0.012075184 | Down |

*Log2 Fold Change reflects changes in hemorrhagic versus ischemic moyamoya disease.

| **Supplementary Table 3. Clinical Characteristics of Patients with Ischemic and Hemorrhagic MMD in CyTOF Cohort.** | | | |
| --- | --- | --- | --- |
| Characteristics | Ischemic MMD (n=20) | Hemorrhagic MMD (n=16) | *p* value |
| Age, y, mean ± SD | 38.20 ± 8.12 | 39.94 ± 5.85 | 0.666 |
| Female/male ratio | 9/11 | 10/6 | 0.335 |
| Clinical features, mean ± SD |  |  |  |
| Heart rate, bpm | 78.80 ± 2.78 | 78.88 ± 4.63 | 0.706 |
| SBP, mmHg | 127.20 ± 10.43 | 132.69 ± 5.82 | 0.069 |
| DBP, mmHg | 77.70 ± 7.38 | 80.94 ± 7.69 | 0.184 |
| BMI, kg/m² | 25.03 ± 3.31 | 26.35 ± 5.84 | 0.924 |
| *RNF213* p.R4810K, n (%) |  |  | 0.672 |
| Wild-type | 16 (80.0) | 14 (87.5) |  |
| Mutant | 4 (20.0) | 2 (12.5) |  |
| Vascular risk factors |  |  |  |
| Hypertension | 9 (45.0) | 3 (18.8) | 0.157 |
| Hypercholesterolemia | 4 (20.0) | 1 (6.3) | 0.355 |
| Diabetes mellitus | 0 (0.0) | 1 (6.3) | 0.444 |
| Current cigarette smoking | 6 (30.0) | 4 (25.0) | 1.000 |
| Current alcohol | 4 (20.0) | 1 (6.3) | 0.355 |
| Suzuki stage, n (%) |  |  | 0.179 |
| ≤ 3 | 13 (65.0) | 6 (37.5) |  |
| > 3 | 7 (35.0) | 10 (62.5) |  |

MMD, moyamoya disease; SD, standard deviation; SBP, systolic blood pressure; DBP, diastolic blood pressure; and BMI, body mass index.

**Supplementary Table 4. Mass Cytometry Antibodies Panel Design.**

| Antigen | Symbol and Mass | Antibody clone | Source |
| --- | --- | --- | --- |
| CD45 | 89Y | HI30 | Fluidigm |
| CD3 | 111Cd | UCHT1 | Fluidigm |
| CCR6 | 141Pr | G034E3 | Fluidigm |
| TLR4 | 142Ce | 76B357.1 | Abcam |
| MyD88 | 143Nd | EPR590(N) | Abcam |
| CCR10 | 144Nd | 314305 | R&D |
| CD16 | 145Nd | 3G8 | Fluidigm |
| CD8 | 146Nd | RPA-T8 | Fluidigm |
| CD11c | 147Sm | Bu15 | Fluidigm |
| CD4 | 148Sm | RPA-T4 | R&D |
| CD25 | 149Sm | BC96 | Fluidigm |
| CD27 | 150Sm | L128 | Fluidigm |
| CD14 | 151Eu | 134620 | R&D |
| IL-12 | 152Gd | 27537 | R&D |
| CD62L | 153Eu | DREG-56 | Fluidigm |
| CCR7 | 154Gd | G043H7 | R&D |
| CD56 | 155Gd | NCAM16.2 | R&D |
| CXCR3 | 156Gd | G025H7 | Fluidigm |
| p-STAT3 | 158Gd | 4/P-STAT3 | Fluidigm |
| Foxp3 | 159Tb | PCH101 | Fluidigm |
| TCRγδ | 160Dy | 11F2 | Fluidigm |
| CD45RA | 162Dy | HI100 | Fluidigm |
| TGFBβ | 163Dy | TW4-6H10 | Fluidigm |
| CD11b | 164Dy | ICRF44 | Fluidigm |
| CD127 | 165Ho | A019D5 | Fluidigm |
| NFκB | 166Er | 532301 | R&D |
| CXCR4 | 167Er | EPR23502-85 | Abcam |
| ERK | 168Er | C-9 | santacruz |
| PD-1 | 169Tm | EH12.2H7 | Fluidigm |
| AKT | 170Yb | 281046 | R&D |
| CD45RO | 171Yb | UCHL1 | Fluidigm |
| CX3CR1 | 172Yb | 528728 | R&D |
| HLA-DR | 173Yb | L243 | R&D |
| p-STAT4 | 174Yb | 38/PSTAT4 | Fluidigm |
| CD19 | 175Lu | HIB19 | R&D |
| IFN-γ | 176Lu | B27 | Biolegend |
| PD-L1 | 209Bi | M1H1 | Fluidigm |


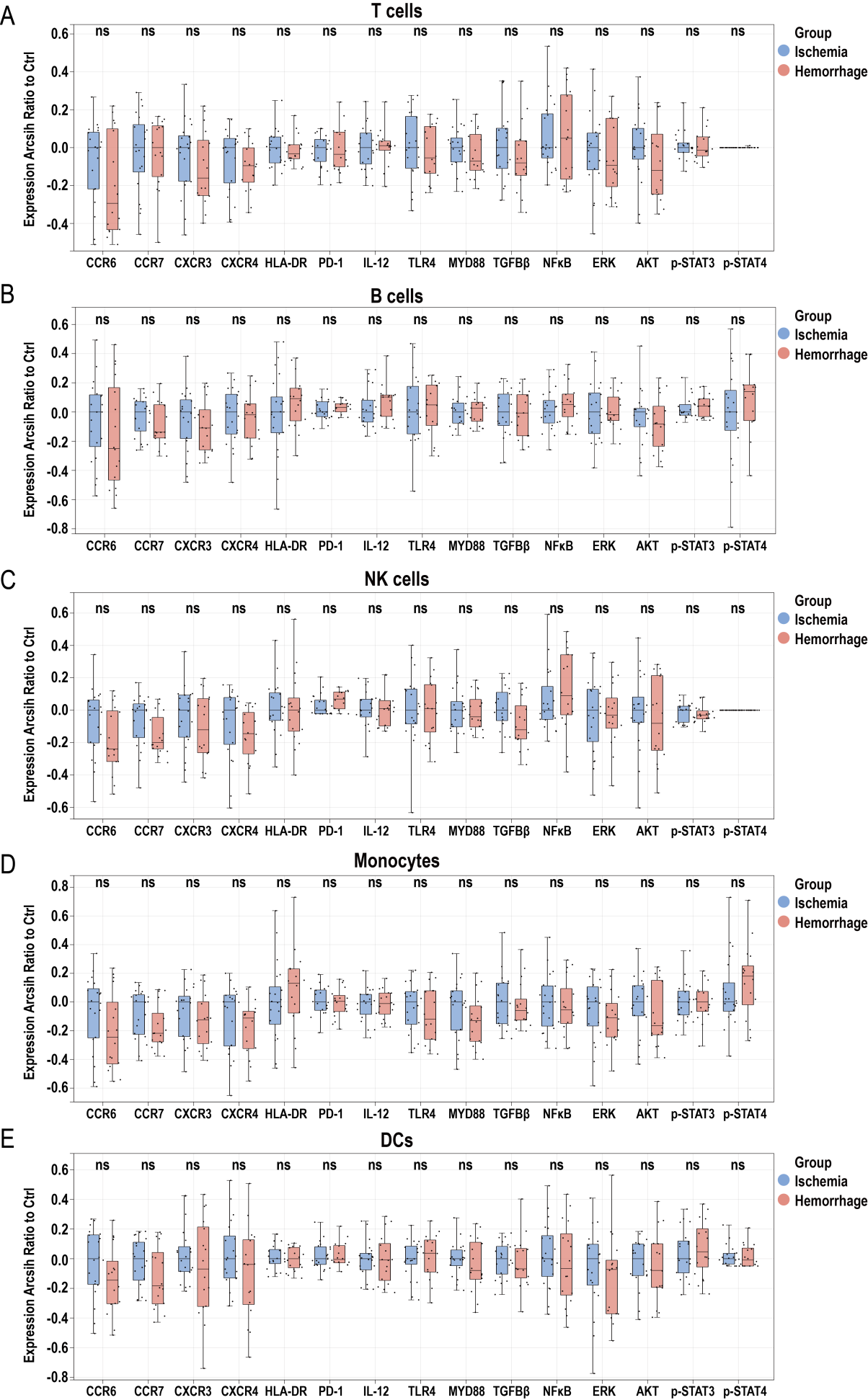


**Figure S1. Molecular Expression Differences Between Ischemic and Hemorrhagic Groups in PBMC Subsets.** (A) T cells. (B) B cells. (C) NK cells. (D) Monocytes. (E) DCs. Significance: ns, p ≥ 0.05.


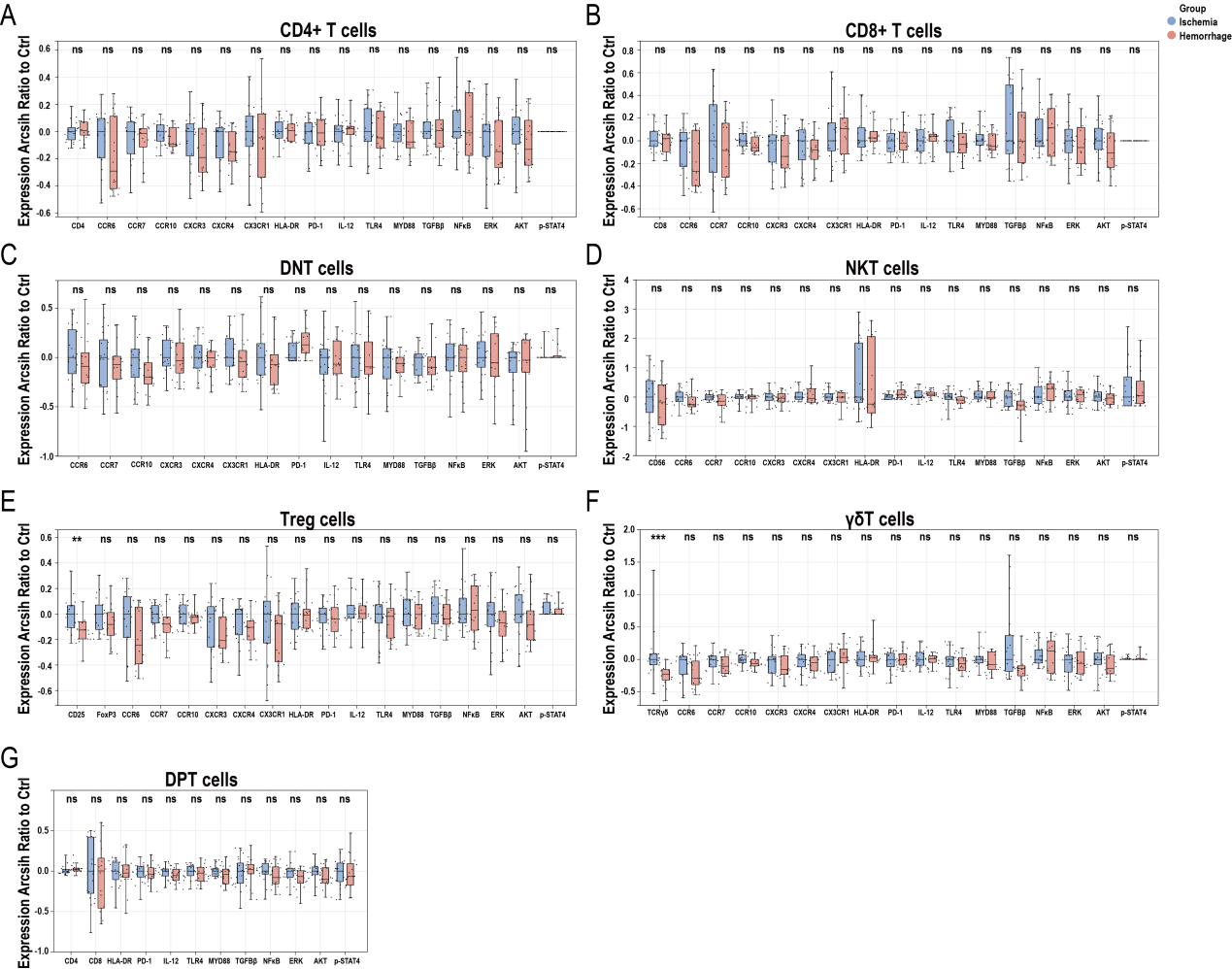


**Figure S2. Molecular Expression Differences Across Seven T Cell Clusters in Ischemic and Hemorrhagic Groups.** (A) CD4^+^ T cells. (B) CD8^+^ T cells. (C) DNT cells. (D) NKT cells. (E) Treg cells. (F) γδT cells. (G) DPT cells. Significance: ns, p ≥ 0.05; **p < 0.01; ***p < 0.001.


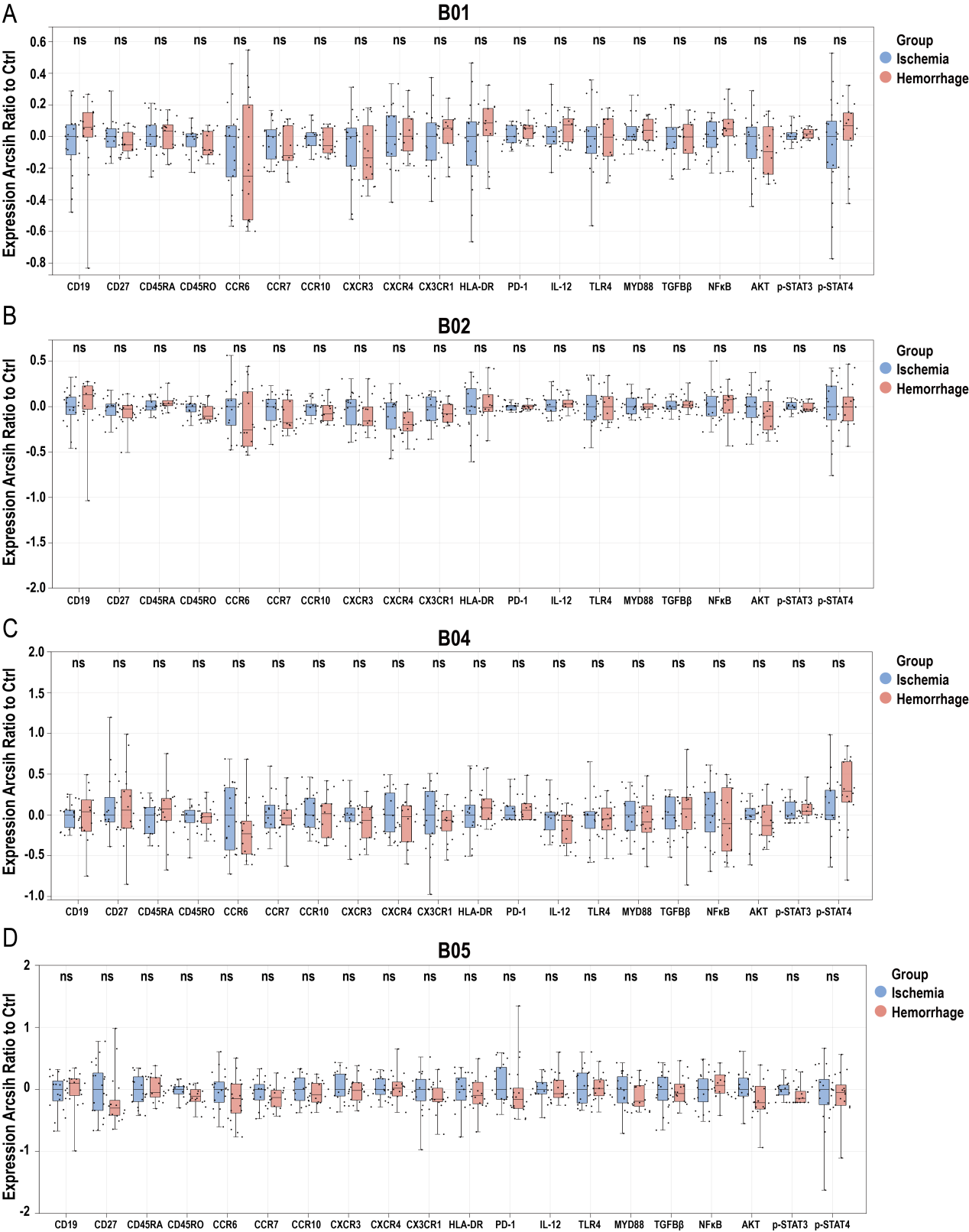


**Figure S3. Molecular Expression Differences Across Four B Cell Clusters in Ischemic and Hemorrhagic Groups.** (A) B01. (B) B02. (C) B04. (D) B05. Significance: ns, p ≥ 0.05.


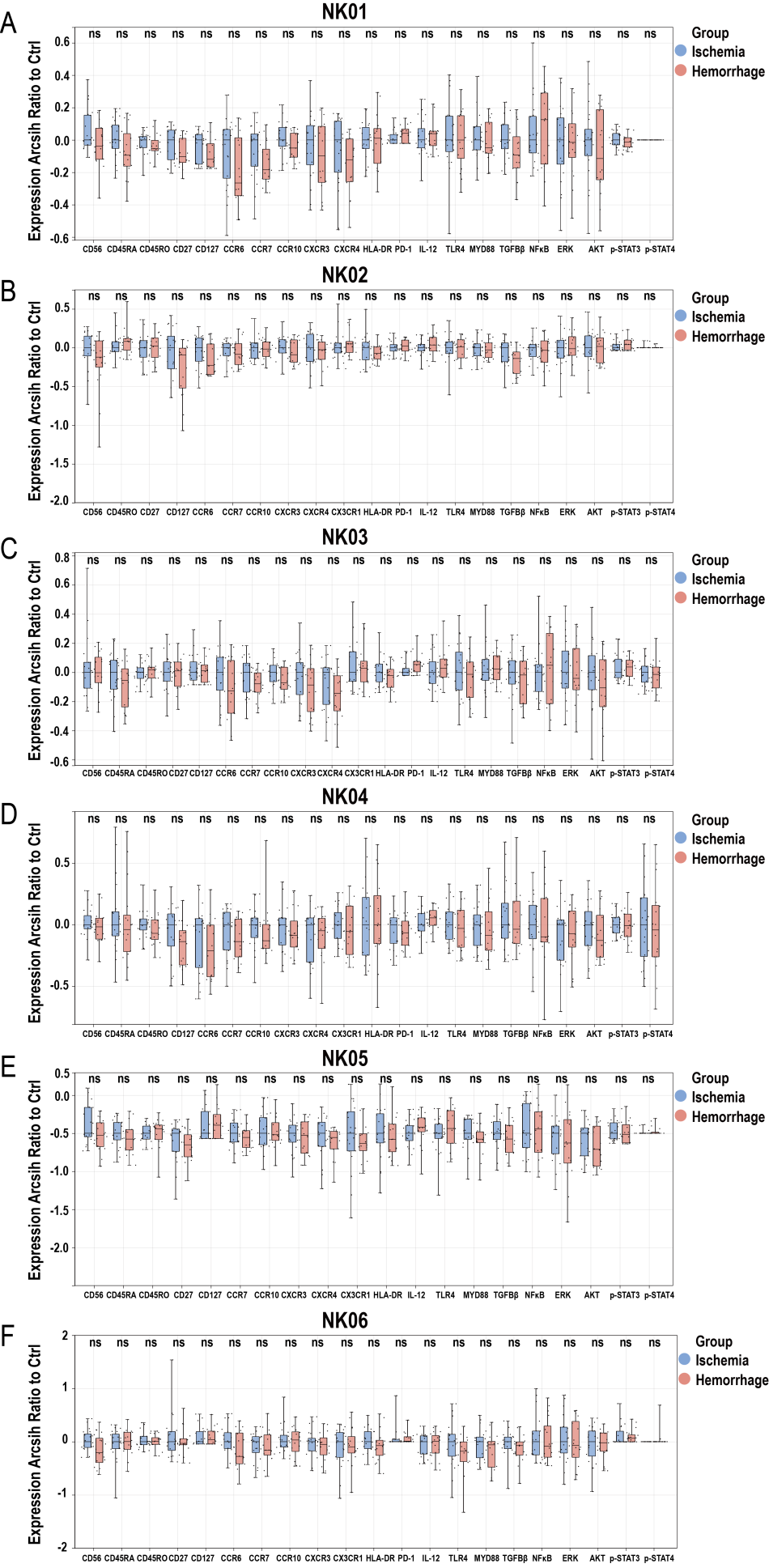


**Figure S4. Molecular Expression Differences Across Six NK Cell Clusters in Ischemic and Hemorrhagic Groups.** (A) NK01. (B) NK02. (C) NK03. (D) NK04. (E) NK05. (F) NK06. Significance: ns, p ≥ 0.05.


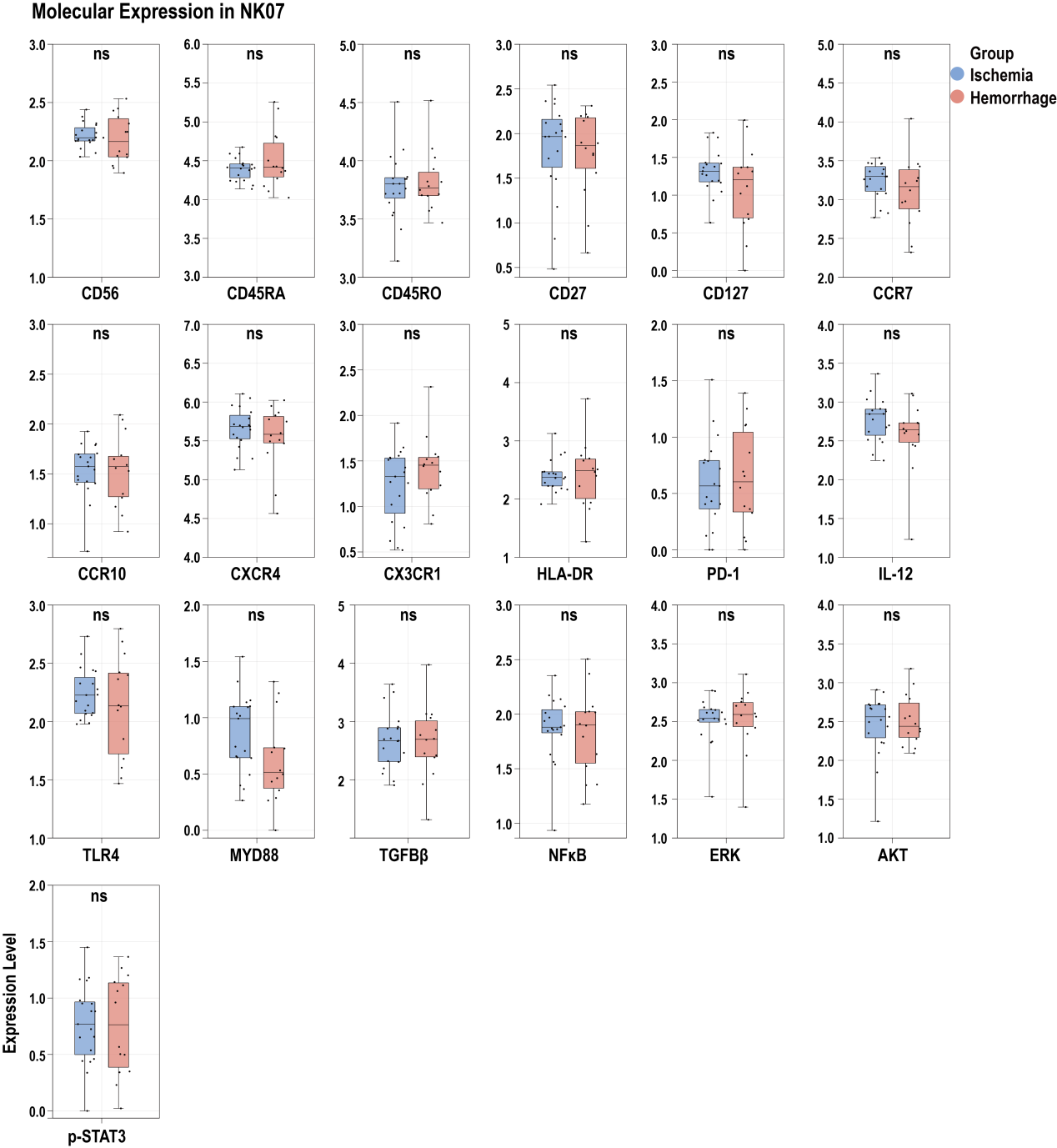


**Figure S5. Molecular Expression in the NK07 Cluster.** Significance: ns, p ≥ 0.05.


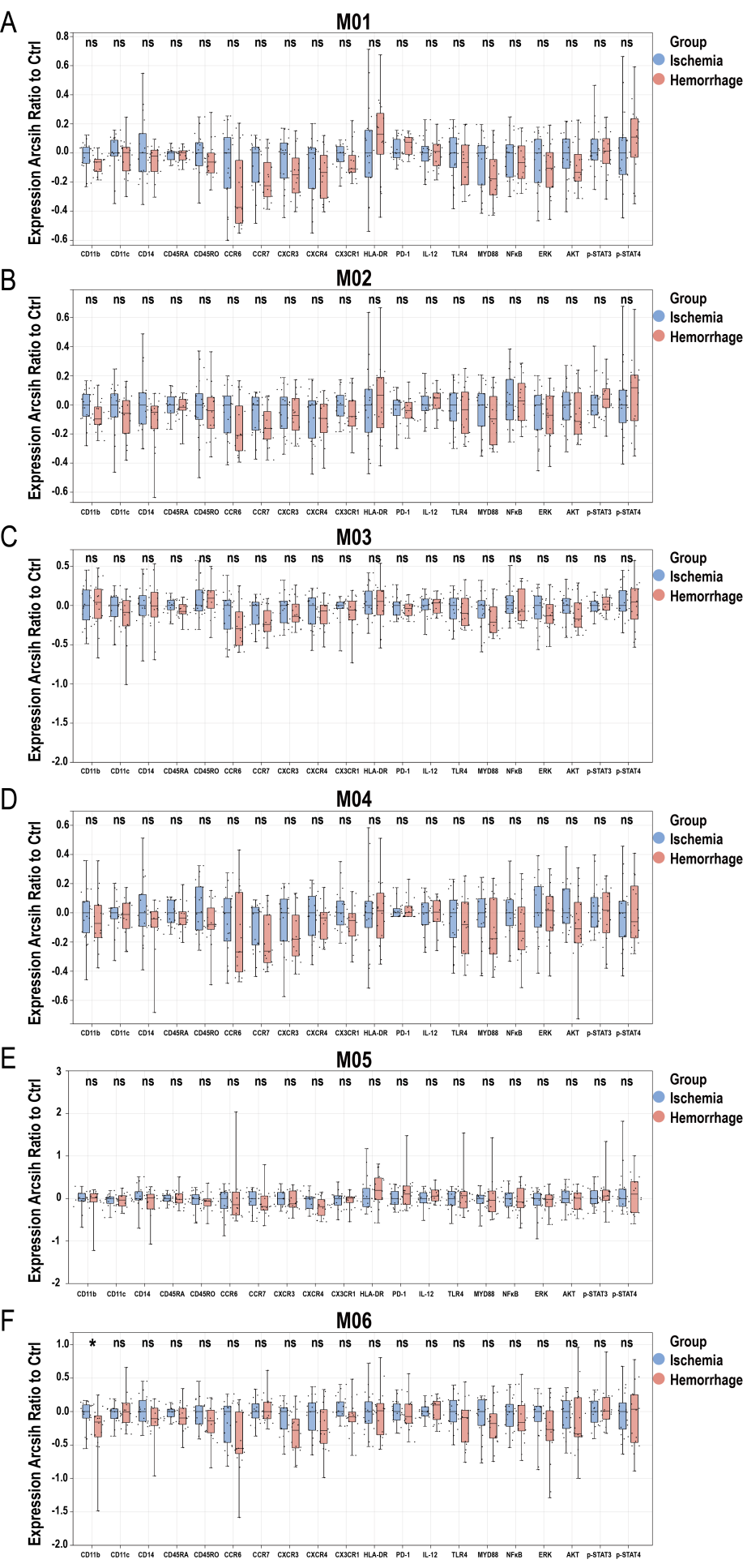


**Figure S6. Molecular Expression Differences Across Six Monocyte Clusters in Ischemic and Hemorrhagic Groups.** (A) M01. (B) M02. (C) M03. (D) M04. (E) M05. (F) M06. Significance: ns, p ≥ 0.05; *p < 0.05.


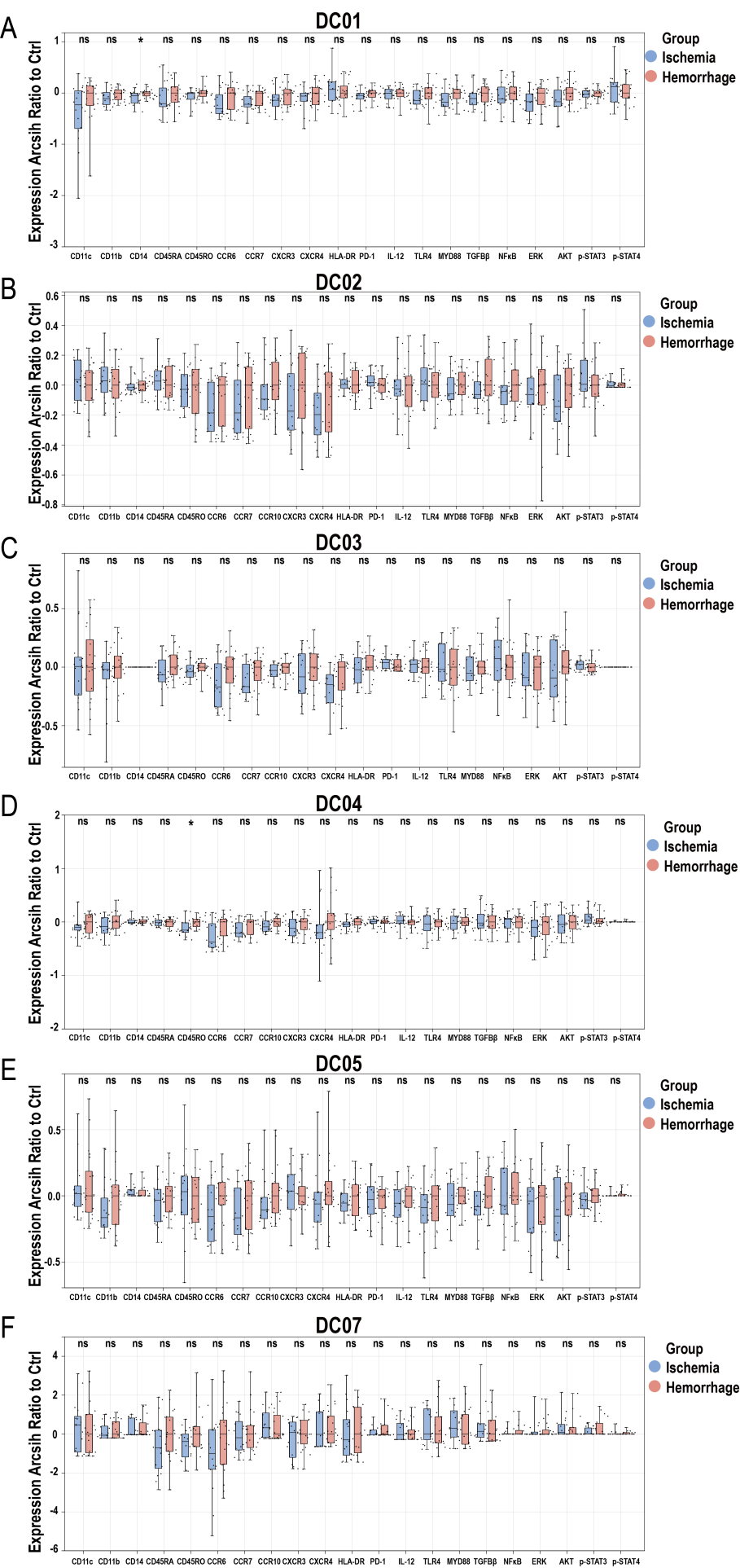


**Figure S7. Molecular Expression Differences Across Six DC Cell Clusters in Ischemic and Hemorrhagic Groups.** (A) DC01. (B) DC02. (C) DC03. (D) DC04. (E) DC06. (F) DC07. Significance: ns, p ≥ 0.05; *p < 0.05.


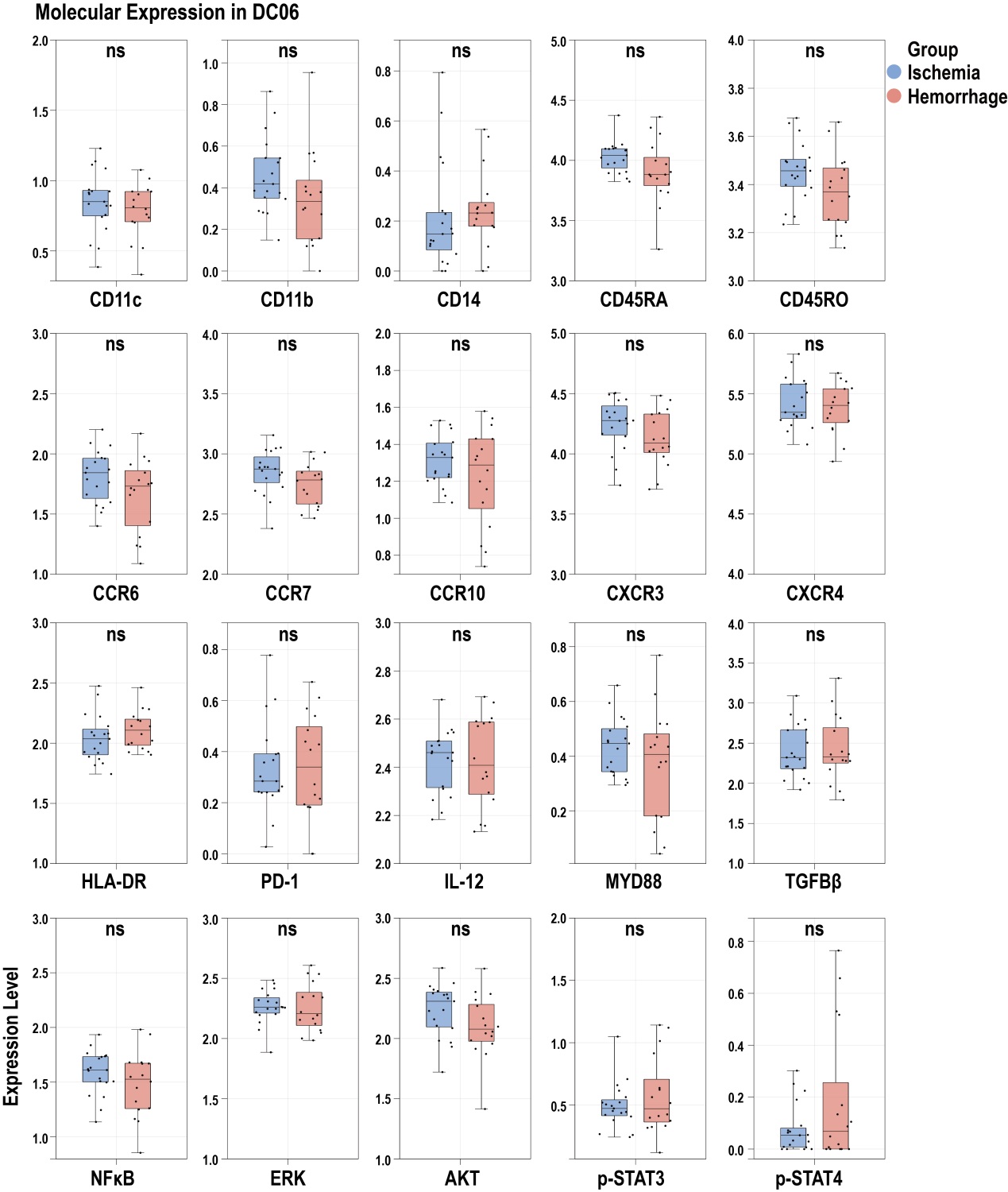


**Figure S8. Molecular Expression in the DC06 Cluster.** Significance: ns, p ≥ 0.05.
